# Zc3h13/Flacc is required for adenosine methylation by bridging the mRNA-binding factor Rbm15/Spenito to the m^6^A machinery component Wtap/Fl(2)d

**DOI:** 10.1101/gad.309146.117

**Published:** 2018-03-01

**Authors:** Philip Knuckles, Tina Lence, Irmgard U. Haussmann, Dominik Jacob, Nastasja Kreim, Sarah H. Carl, Irene Masiello, Tina Hares, Rodrigo Villaseñor, Daniel Hess, Miguel A. Andrade-Navarro, Marco Biggiogera, Mark Helm, Matthias Soller, Marc Bühler, Jean-Yves Roignant

**Affiliations:** 1 Friedrich Miescher Institute for Biomedical Research, 4058 Basel, Switzerland;; 2 University of Basel, Basel 4002, Switzerland;; 3 Institute of Molecular Biology, 55128 Mainz, Germany;; 4 School of Life Science, Faculty of Health and Life Sciences, Coventry University, Coventry CV1 5FB, United Kingdom;; 5 School of Biosciences, College of Life and Environmental Sciences, University of Birmingham, Edgbaston, Birmingham B15 2TT, United Kingdom;; 6 Institute of Pharmacy and Biochemistry, Johannes Gutenberg University of Mainz, 55128 Mainz, Germany;; 7 Bioinformatics Core Facility, Institute of Molecular Biology, 55128 Mainz, Germany;; 8 Swiss Institute of Bioinformatics, Basel 4058, Switzerland;; 9 Laboratory of Cell Biology and Neurobiology, Department of Animal Biology, University of Pavia, Pavia 27100, Italy;; 10 Faculty of Biology, Johannes Gutenberg University of Mainz, 55128 Mainz, Germany

**Keywords:** Zc3h13, Flacc, m^6^A, methyltransferase complex, RNA modifications, sex determination

## Abstract

*N*^6^-methyladenosine (m^6^A) is the most abundant mRNA modification in eukaryotes, playing crucial roles in multiple biological processes. m^6^A is catalyzed by the activity of methyltransferase-like 3 (Mettl3), which depends on additional proteins whose precise functions remain poorly understood. Here we identified Zc3h13 (zinc finger CCCH domain-containing protein 13)/Flacc [Fl(2)d-associated complex component] as a novel interactor of m^6^A methyltransferase complex components in *Drosophila* and mice. Like other components of this complex, Flacc controls m^6^A levels and is involved in sex determination in *Drosophila*. We demonstrate that Flacc promotes m^6^A deposition by bridging Fl(2)d to the mRNA-binding factor Nito. Altogether, our work advances the molecular understanding of conservation and regulation of the m^6^A machinery.

## Abstract (precis)

In this study, Knuckles et al. identified Flacc/Zc3h13 as a novel interactor of m^6^A methyltransferase complex components in *Drosophila* and mice. They show that Flacc promotes the recruitment of the methyltransferase to mRNA by bridging Fl(2)d to the mRNA-binding factor Nito, providing novel insights into the conservation and regulation of the m^6^A machinery.

In the past years, *N*^6^-methyladenosine (m^6^A) RNA has emerged as an abundant and dynamically regulated modification throughout the transcriptome (Dominissini et al. 2012; Meyer et al. 2012). m^6^A affects almost every stage of mRNA metabolism, and its absence is associated with various defects in meiosis, embryonic stem cell (ESC) differentiation, DNA repair, circadian rhythm, neurogenesis, dosage compensation, and sex determination (for a recent review, see Roignant and Soller 2017; Zhang et al. 2017). Alteration of m^6^A levels also promotes glioblastoma progression and is linked to poor prognosis in myeloid leukemia (Barbieri et al. 2017; Cui et al. 2017; Jaffrey and Kharas 2017; Kwok et al. 2017; Li et al. 2017; Vu et al. 2017; Weng et al. 2018).

Formation of m^6^A is catalyzed by the activity of methyltransferase-like 3 (METTL3; also called MT-A70) (Bokar et al. 1997), which physically interacts with METTL14 (Liu et al. 2014; Ping et al. 2014; Schwartz et al. 2014; Wang et al. 2014), Wilms tumor 1-associated protein (WTAP) (Zhong et al. 2008; Agarwala et al. 2012), Vir-like m^6^A methyltransferase-associated (KIAA1429/VIRMA) (Schwartz et al. 2014), and RNA-binding motif 15 (RBM15) and its paralog, RBM15B (Patil et al. 2016). *Drosophila* has corresponding homologs Mettl3, Mettl14, Fl(2)d, Virilizer (Vir), and Spenito (Nito) (Lence et al. 2017). Recent crystal structural studies investigated the molecular activities of the two predicted methyltransferases METTL3 and METTL14 (Sledz and Jinek 2016; Wang et al. 2016a,b). Only METTL3 was shown to contain the catalytic activity and form a stable heterodimer with METTL14, which was required to enhance METTL3 enzymatic activity by binding substrate RNA and positioning the methyl group for transfer to adenosine. In addition, WTAP [Fl(2)d] ensures the stability and localization of the heterodimer to nuclear speckles (Ping et al. 2014; Lence et al. 2016). VIRMA (Vir) is essential for m^6^A deposition, but its molecular function is currently unknown. Last, RBM15 and RBM15B (Nito) have been suggested to recruit the methyltransferase complex to its target transcripts via direct binding to U-rich sequences on mRNA. In humans, this function is important to control m^6^A promoted X-chromosome inactivation via *XIST*-mediated transcriptional repression (Patil et al. 2016). In *Drosophila*, Nito promotes m^6^A function in the sex determination and dosage compensation pathways (Lence et al. 2016).

To date, it is unknown how Nito in *Drosophila* interacts with other members of the methyltransferase writer complex to ensure their recruitment to mRNA targets. Although, in human cells, RBM15/15B were reported to interact with METTL3 in a WTAP-dependent manner (Patil et al. 2016), it is unclear whether this interaction is conserved in other organisms. In order to address these questions, we performed interactome analyses from *Mus musculus* and *Drosophila melanogaster* cell extracts using Rbm15 and Nito as bait, respectively. We identified mouse zinc finger CCCH domain-containing protein 13 (Zc3h13) and its fly homolog, CG7358, which we named Fl(2)d-associated complex component (Flacc), as novel interactors of the m^6^A writer machinery. A lack of these proteins dramatically reduces m^6^A levels in both organisms. Consistent with the role of m^6^A in sex determination in *Drosophila*, Flacc depletion results in aberrant splicing of *Sex lethal* (*Sxl*) and leads to transformations of female into male-like structures. Moreover, we demonstrate that Flacc interacts with Nito and Fl(2)d and serves as an adaptor between these two proteins, thereby stabilizing the complex and promoting m^6^A deposition on mRNA.

## Results

### Zc3h13 interacts with the m^6^A machinery

**Figure 1.**
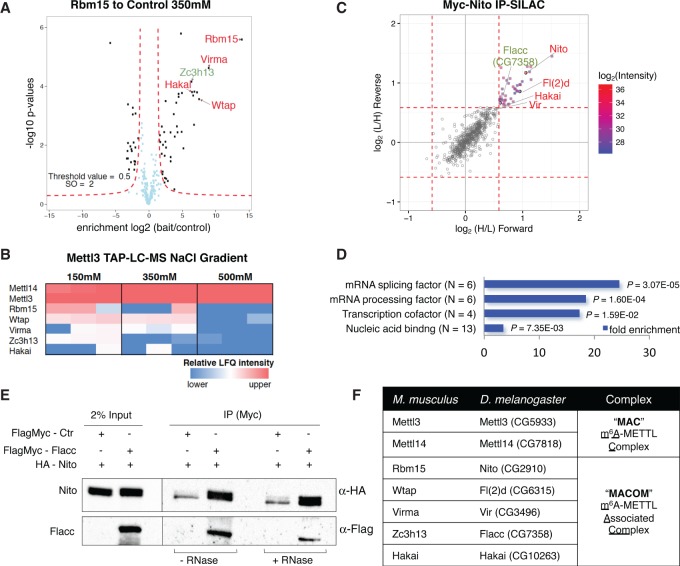
Zc3h13/Flacc interacts with the m^6^A machinery. (*A*) TAP-LC-MS/MS of endogenously Flag-Avi-tagged Rbm15 mESCs. Parental cells were used as background control, and proteins were purified in the presence of 350 mM NaCl. Highlighted in the volcano plot are enriched proteins previously identified as Mettl3 interactors (red) as well as Zc3h13 (green). (*B*) Heat map comparing relative label-free quantification (LFQ) intensities of selected Mettl3-bound proteins across increasing NaCl concentrations. Statistical analysis was done with Perseus (see the Materials and Methods for details). MS raw data were deposited in ProteomeXchange. (*C*,*D*) Stable isotope labeling of amino acids in cell culture (SILAC) coupled to MS analysis using Nito-Myc as bait. Scatter plot of normalized forward versus inverted reverse experiments plotted on a log_2_ scale. The threshold was set to a 1.5-fold enrichment (red dashed line). Proteins in the *top right* quadrant of *C* are enriched in both replicates. Gene ontology (GO) term analysis (Tyanova et al. 2016) for enriched proteins is shown in *D*. (*E*) Coimmunoprecipitation experiments were carried out with lysates prepared from S2R^+^ cells transfected with FlagMyc-Flacc and HA-Nito. In control lanes, S2R^+^ cells were transfected with FlagMyc alone and an identical HA-containing protein. Extracts were immunoprecipitated with Myc antibody and immunoblotted using Flag and HA antibodies. Two percent of input was loaded. The same experiment was repeated in the presence of RNaseT1. Nito and Flacc interact with each other in an RNA-independent manner. (*F*) Table representing orthologous proteins of the m^6^A–METTL complex (MAC) and the m^6^A-METTL-associated complex (MACOM) in mice and flies.

In our recent work, we identified Nito as a novel interactor of the m^6^A methyltransferase complex (Lence et al. 2016). Because the role of the mouse Nito homolog protein Rbm15 appears to be evolutionarily conserved in regard to m^6^A deposition (Patil et al. 2016), we sought to identify interaction partners to obtain further insights into Rbm15's function. To this end, we tagged endogenous Rbm15 with the Flag-Avi tag using CRISPR–Cas9 genome editing in mouse ESCs (mESCs) that express the bacterial *BirA* ligase (Supplemental Fig. S1A, B; Flemr and Buhler 2015). Subsequently, we performed tandem affinity purification (TAP) coupled to liquid chromatography and mass spectrometry (TAP-LC-MS). We found that Rbm15 copurifies with Wtap, Virma, and Hakai (Fig. 1A) under stringent conditions (350 mM NaCl), demonstrating that these proteins stably interact with each other. Hakai was found recently to interact with other subunits of the m^6^A methyltransferase complex in plants (Ruzicka et al. 2017). Interestingly, we also observed Zc3h13 among the top hits. Although it was reported to interact with WTAP in human cells, it has not been linked previously to adenosine methylation (Horiuchi et al. 2013; Wan et al. 2015).

Previous work suggested that the interaction of the heterodimer Mettl3/14 with Wtap, Virma, and Rbm15 is important to guide the methylation complex to its targets and correctly methylate mRNA (Ping et al. 2014; Schwartz et al. 2014; Patil et al. 2016). To test whether the Zc3h13-containing protein complex described above interacts with Mettl3/14 in mice, we also endogenously tagged Mettl3 with the Flag-Avi tag in mESCs (Supplemental Fig. S1A, B) and performed TAP-LC-MS. Consistent with previous reports, we found that Mettl3 copurifies with Mettl14, Wtap, Virma, Rbm15, and Hakai (Fig. 1B; Supplemental Fig. S2A). Importantly, we also recovered peptides from Zc3h13 (Fig. 1B; Supplemental Fig. S2A). Whereas these interactions resisted 350 mM NaCl, only the Mettl3/Mettl14 interaction remained at 500 mM NaCl (Fig. 1B). These results indicate the existence of two stable protein complexes (Mettl3/Mettl14 and Rbm15/Zc3h13/Wtap/Virma/Hakai), which we refer to as the m^6^A–METTL complex (MAC) and the m^6^A-METTL-associated complex (MACOM), respectively.

To gain further insight into the relative amounts of MAC to MACOM, we performed intensity-based absolute quantification (iBAQ) analysis on TAP-LC-MS data from endogenously tagged Mettl3. We observed comparable stoichiometry between the bait (Mettl3) and Mettl14. In contrast, Wtap and other MACOM components were <1% abundant compared with Mettl3 and Mett14 (Supplemental Fig. S2B), an observation that we interpret as a sign of a weak and/or short-lived interaction. Alternatively, the abundance of MAC bound to MACOM could be very scarce relative to the level of each independent complex. Regardless of the precise mechanism, because components of both complexes are required to install m^6^A, we propose that MAC and MACOM interact with each other in order to deposit the methylation.

### The *Drosophila* Zc3h13 homolog Flacc interacts with components of the m^6^A methyltransferase complex

To address whether Nito interacts with the same set of proteins that we identified in mice, we took an approach very similar to that described above using extracts from *Drosophila* S2R^+^ cells. We used stable isotope labeling of amino acids in cell culture (SILAC)-based quantitative proteomics. A Myc-tagged version of Nito was used to perform coimmunoprecipitation experiments from S2R^+^ cells. In total, we identified 40 factors that showed >1.5-fold enrichment in the Nito-Myc precipitate in comparison with control cells transfected with Myc alone (Fig. 1C; Supplemental Table 1). In agreement with mouse Rbm15 proteomic analysis, the homolog of Wtap in *Drosophila* Fl(2)d, was among the top candidates. We also found the previously reported m^6^A writers Vir and Hakai (refer to Fig. 1F for *M. musculus* and *D. melanogaster* nomenclature of m^6^A factors). We observed an overall enrichment for mRNA-binding proteins (Fig. 1D) and, importantly, Flacc, which is the closest homolog of Zc3h13. To confirm the interaction of this protein with Nito, we generated tagged proteins and performed coimmunoprecipitation assays. These experiments confirmed that Flacc interacts with Nito and that this occurs in an RNA-independent manner (Fig. 1E). To verify that Flacc interacts with other components of the m^6^A methyltransferase complex, we immunoprecipitated Flacc-Myc and tested for the presence of Vir and Fl(2)d. As shown in Supplemental Figure S3, A and B, Flacc interacts with both proteins independently of RNA, indicating that it might be a novel regulator of the m^6^A pathway. In contrast to mouse Zc3h13 (see below), however, Flacc does not contain a zinc finger motif (Supplemental Fig. S4).

### Zc3h13/Flacc regulates the m^6^A pathway

**Figure 2.**
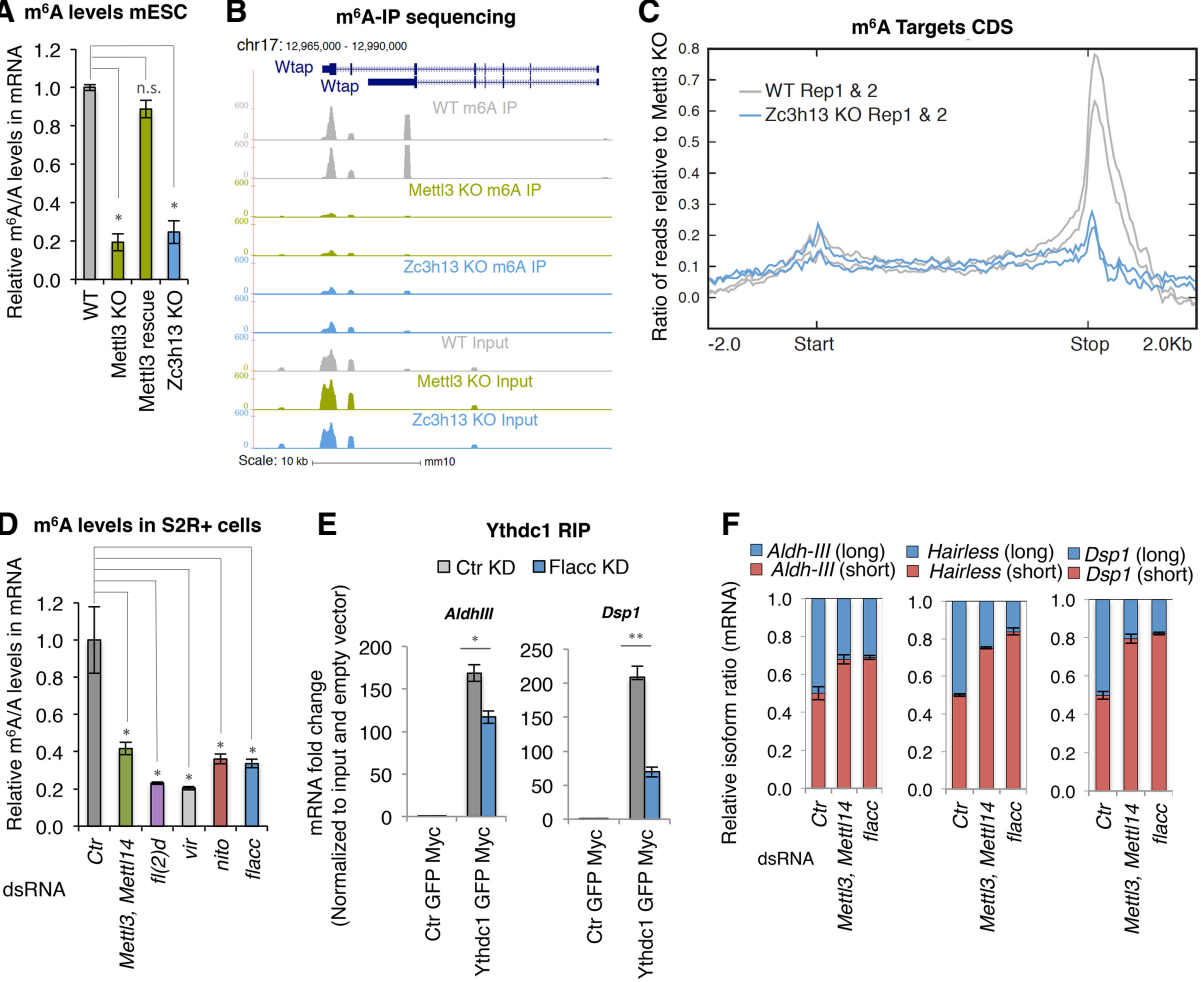
Flacc/Zc3h13 regulates the m^6^A pathway. (*A*) LC-MS/MS quantification of m^6^A levels in mRNA extracts from wild-type mESCs, *Mettl3* knockout and *Mettl3* knockout plasmid rescue, and *Zc3h13* knockout cells. The mean of two biological replicates and three independent measurements is shown. Errors bars indicate standard deviation (SD). (*) *P* < 0.01; (n.s.) not significant, Student's *t*-test. (*B*) University of California at Santa Cruz genome browser shots of m^6^A immunoprecipitation profiles of RNA isolated from *Mettl3* knockout, *Zc3h13* knockout, and wild-type cells and input samples for each genetic background at the Wtap-encoding locus. Scale is mapped reads in 100-base-pair (bp) bins normalized to mean library size. (*C*) Metaplot depicting reads from m^6^A immunoprecipitations at target genes (defined as genes overlapping or within 500 bp of MACS-identified peaks of m^6^A immunoprecipitation/input in wild-type cells) aligned to the coding sequence (“start” and “stop” refer to translation start and stop, respectively). (*D*) LC-MS/MS quantification of m^6^A levels in either control samples or mRNA extracts depleted for the indicated proteins in S2R^+^ cells. The bar chart shows the mean of three biological replicates and three independent measurements. Errors bars indicate SD. (*) *P* < 0.01, Student's *t*-test. Knockdown of the indicated proteins significantly reduces m^6^A levels. (*E*) Fold enrichment of m^6^A-regulated transcripts (A*ldh-III* and *Dsp1*) over input in Myc-Ythdc1 RIP after control or Flacc depletion. The bar chart shows the mean of three biological replicates. Errors bars indicate SD. (*) *P* < 0.01; (**) *P* < 0.001, Student's *t*-test. Loss of Flacc affects Ythdc1 binding. (*F*) Relative isoform quantification of m^6^A-regulated genes (A*ldh-III*, *Hairless*, and *Dsp1*) upon depletion of the indicated components. Flacc is required for m^6^A-dependent splicing events.

To test whether Zc3h13 is necessary for adenosine methylation in mice, we measured global m^6^A levels by LC-MS/MS in *Zc3h13* knockout mESCs (Supplemental Fig. S1C). We found an 80% reduction of m^6^A, similar to isogenic *Mettl3* knockout mESCs (Fig. 2A). Consistent with a global reduction in m^6^A levels, *Zc3h13* knockout cells displayed a drastic change in morphology, reminiscent of *Mettl3* knockout, with loss of dome-shaped colony formation and an overall increase in cell size (data not shown). In addition, we performed m^6^A RNA immunoprecipitation coupled to deep sequencing (m^6^A-RIP-seq) on oligo-dT-selected mRNA from wild-type, *Mettl3* knockout, and *Zc3h13* knockout mESCs. As observed with *Mettl3* knockout cells, ablation of *Zc3h13* resulted in a drastic reduction of m^6^A enrichment, particularly at the 3′ end of target mRNAs (Fig. 2B,C). Hence, we conclude that Zc3h13 is essential for m^6^A installation in mESCs. To investigate evolutionary conservation of this activity in *Drosophila*, we quantified m^6^A levels using LC-MS/MS analysis upon Flacc depletion in *Drosophila* S2R^+^ cells. Similar to the reduction observed upon knockdown of other m^6^A components, depletion of Flacc also resulted in strongly reduced m^6^A levels (Fig. 2D; Supplemental Fig. S5A). This was not due to an indirect effect on expression of other components of the methyltransferase complex (Supplemental Fig. S5B–D). In agreement with decreased m^6^A levels, we found that binding of the reader protein Ythdc1 to its target transcripts was reduced in the absence of Flacc (Fig. 2E; Supplemental Fig. S3C,D). Together, these results demonstrate that Zc3h13 and its *Drosophila* ortholog, Flacc, are novel and essential components of the m^6^A pathways in mice and flies.

### Flacc is required for pre-mRNA splicing

**Figure 3.**
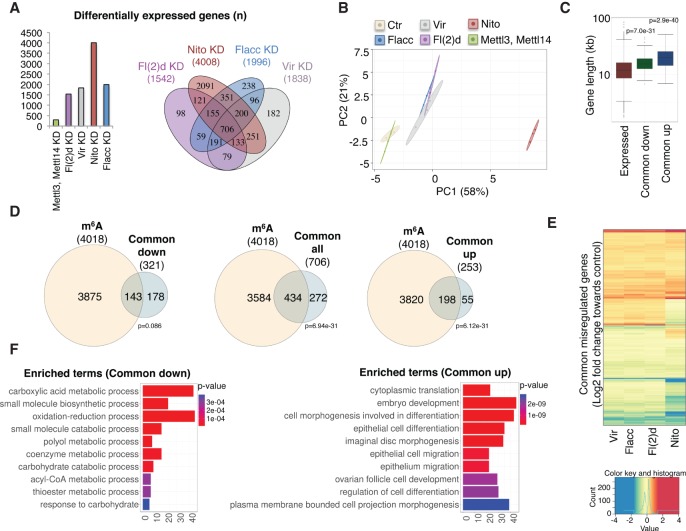
Flacc regulates common transcripts with other components of the m^6^A complex. (*A*) Number of differentially expressed genes (5% false discovery rate [FDR]) upon knockdown of the indicated proteins (*left*) and common differentially expressed targets regulated by components of MACOM (*right*). (*B*) Scatter plot of the first two principal components of a principal component analysis (PCA) of the 500 most variable genes in all conditions. The biological replicates are indicated in the same color, with elliptical areas representing the SD of the two depicted components. (*C*) Gene length distribution for genes tested in the differential expression analysis and the differential expressed genes up-regulated or down-regulated in all conditions. The distributions were tested for difference using the Kolmogorov-Smirnov test. (*D*) Overlap between common up, down, or all differentially expressed genes and genes annotated to have m^6^A-modified transcripts (according to methylation individual nucleotide resolution cross-linking immunoprecipitation data from Kan et al. 2017). The significance of the overlap was tested using a hypergeometric test. (*E*) Fold change (log_2_) expression of commonly misregulated genes. The heat map is clustered according to rows and columns. The color gradient was adjusted to display the 1% lowest/highest values within the most extreme color (lowest values as the darkest blue and highest values as the darkest red). (*F*) The GO term analysis of common up-regulated and down-regulated genes performed using the package ClusterProfiler. The top 10 GO terms are displayed.

To further corroborate Flacc as a bona fide m^6^A writer, we tested whether it was required to control m^6^A splicing-related events. As reported previously, splicing of several transcripts, including *AldhIII*, *Dsp1*, and *Hairless,* is dependent on the m^6^A pathway (Lence et al. 2016). Remarkably, depletion of Flacc affected all of those transcripts (Fig. 2F; Supplemental Fig. S5E). We next expanded this analysis to a transcriptome-wide level, which revealed that depletion of Flacc in S2R^+^ cells leads to changes in gene expression and splicing that substantially overlap with changes observed upon knockdown of other m^6^A writers (Fig. 3A; Supplemental Fig. S6A; Supplemental Tables 3–8). In particular, the Flacc-depleted transcriptome clusters very closely with Fl(2)d- and Vir-depleted transcriptomes (Fig. 3B). Notably, Nito depletion induced greater changes and poorly clustered with the others, suggesting that Nito might be pleiotropic. Regardless of Nito's potential role in other pathways, common misregulated genes among components of the MACOM are larger than the average gene size (Fig. 3C) and are significantly methylated (61.5%; *P* = 6.94 × 10^−31^) (Fig. 3D). Importantly, differentially expressed genes generally change in the same direction upon the different knockdowns, confirming that MACOM components belong to the same complex and share similar functions (Fig. 3E). We noticed that common up-regulated genes tend to be larger (*P* = 2.9 × 10^−40^) and more methylated compared with down-regulated ones (78.2% [*P* = 6.12 × 10^−31^] vs. 44.5% [*P* = 0.086]) (Fig. 3C,D). Up-regulated genes were enriched for processes involved in embryonic development as well as epithelial cell differentiation and migration (Fig. 3F). Thus, it is possible that down-regulated genes, which are mostly enriched for metabolic processes, are affected indirectly (Fig. 3F).

We next performed similar analysis with respect to splicing changes. We found that knockdown of each of the known m^6^A writer components, including Flacc, resulted in an increase of both alternative 5′ splice site usage and intron retention (Supplemental Fig. S6A,B). Moreover, most common misspliced transcripts upon knockdown of MACOM components are methylated (82.2%; *P* = 1.3 × 10^−8^), show similar splicing defects, and are enriched for neuronal processes, which is consistent with our previous findings (Supplemental Fig. S6C–E; Lence et al. 2016). Of note, knockdown of Mettl3/Mettl14 generally produces less effect compared with knockdown of MACOM components. This may be explained by residual m^6^A activity upon knockdown of the methyltransferases. Alternatively, MACOM components may have additional functions beyond m^6^A activity (see also the Discussion).

### Flacc subcellular localization and expression through development

To further investigate the role of Flacc in *Drosophila*, we examined its subcellular localization as well as its developmental expression profile. We observed that Flacc is strictly localized in the nucleus in S2R^+^ cells (Supplemental Fig. S7A) and that its transcript is broadly expressed during embryogenesis but shows enrichment in the neuroectoderm (Supplemental Fig. S7B). Overall, *flacc* mRNA follows the same distribution as transcripts of other subunits of the methyltransferase complex (Lence et al. 2016) and as m^6^A levels in mRNA. An exception is the stage of maternal-to-zygotic transition (2 h after fertilization), where a boost of *flacc* expression is observed while m^6^A is rapidly decreasing (Supplemental Fig. S7C), suggesting that Flacc might have an additional function in early embryogenesis.

### Flacc is required for sex determination and dosage compensation via *Sxl* alternative splicing

**Figure 4.**
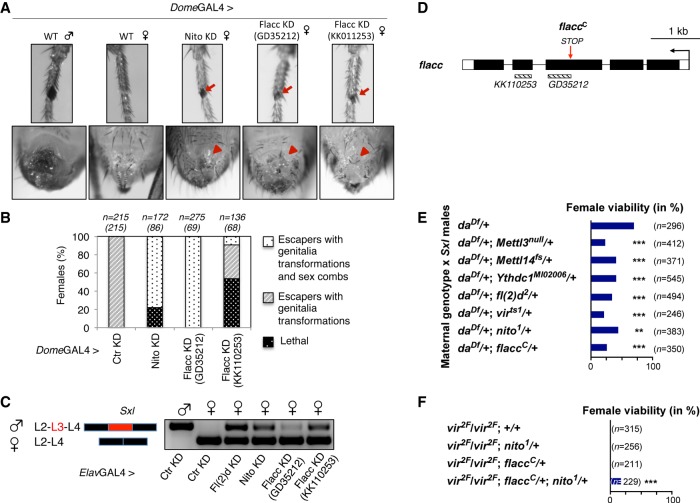
Flacc is required for sex determination via control of *Sxl* alternative splicing. (*A*,*B*) *dome*-GAL4-driven expression of shRNA or dsRNA in genital discs and first pair of leg discs against Nito or Flacc, respectively. (*Top*) Forelegs of a wild-type male fly and female flies depleted for Nito or Flacc show the appearance of male-specific sex comb bristles (red arrow). (*Bottom*) Depletion of Nito or Flacc results in transformations of female genitalia and loss of vaginal bristles (red arrowhead). (*B*) Quantification of female survival and transformations in escapers upon depletion of Nito or Flacc using the *dome*-GAL4 driver. (*n*) The number of analyzed flies with the expected number of escapers in brackets. Depletion of Nito and Flacc results in a high level of transformation in female genitalia and the appearance of male-specific sex combs on forelegs. (*C*) Semiquantitative RT–PCR analysis of *Sxl* isoforms in male and female heads from flies depleted for Fl(2)d, Nito, or Flacc, respectively, using the *Elav*GAL4 driver. Inclusion of male-specific exon L3 is observed in flies lacking m^6^A components. (*D*) The *flacc* locus (*flacc*^*C*^) with a premature stop codon at amino acid Leu730. Sites of dsRNA fly lines KK110253 and GD35212 are shown *below* gene loci. (*E*) Viability of female flies from a cross of the indicated genotypes mated with *Sxl*^*7BO*^ males. The loss of one copy of *flacc* significantly reduces female survival in a genetic background where one copy of *Sxl* and *da* are absent. The same compromised survival is observed for other m^6^A components [*Mettl3*, *Mettl14*, *Ythdc1*, *fl(2)d*, *vir*, and *nito*]. Viability was calculated from the numbers of females compared with males, and statistical significance was determined by a χ^2^ test (Graphpad Prism). (*F*) The viability of female flies with homozygous *vir2F* mutation can be rescued by the loss of a single copy of *flacc* and *nito*. Viability was calculated from the numbers of homozygous *vir2F* females compared with heterozygous balancer-carrying siblings, and statistical significance was determined by a χ^2^ test (Graphpad Prism).

Components of the m^6^A machinery were shown previously to affect sex determination and dosage compensation in *Drosophila* via the control of *Sxl* alternative splicing (Haussmann et al. 2016; Lence et al. 2016; Kan et al. 2017). To address whether Flacc bears similar functions, we depleted its products by expressing corresponding dsRNA in both the legs and genital discs using the *dome*-GAL4 driver. Strikingly, these females displayed clear transformations into male structures, as shown previously for Nito (Fig. 4A; Yan and Perrimon 2015). This is illustrated by the appearance of sex combs in the forelegs of females that were depleted for Flacc. The phenotype was observed in ∼20% of females examined (Fig. 4B). Using a dsRNA that targets a distinct region of *flacc* (GD35212), the penetrance was even increased to all female escapers (Fig. 4B; Supplemental Fig. S7D). Furthermore, typical female external structures, such as vaginal bristles, were absent on the same individuals (Fig. 4A). Altogether, these data indicate that Flacc plays a major role in the control of sex determination in flies.

To address how Flacc affects sex determination, we tested whether alternative splicing of *Sxl*, the master regulator of sex determination and dosage compensation, was affected. RNA extracts from fly heads depleted by RNAi for Fl(2)d, Nito, or Flacc were subjected to reverse transcription followed by PCR using primers spanning the common exons 2 and 4. While the male-specific exon 3 is absent in control female heads, it was clearly included upon the loss of components of the m^6^A machinery, including Flacc (Fig. 4C). This experiment indicates that Flacc regulates sex determination and dosage compensation via *Sxl* alternative splicing, as shown previously for other m^6^A writers.

To confirm the effect of Flacc on sex determination via *Sxl* alternative splicing observed when using RNAi, we analyzed a lethal *flacc* mutant allele harboring a stop codon at amino acid 730 (Fig. 4D). Reducing one copy of m^6^A components [*Mettl3*, *Mettl14*, *fl(2)d*, *vir*, *nito*, or *Ythdc1*] in a sensitized background (heterozygous for *Sxl* and *daughterless*) significantly alters female viability (Fig. 4E). We showed previously for the *Mettl3* allele that this is due to activation of dosage compensation in females (Haussmann et al. 2016). Consistent with its role in *N*^*6*^-adenosine methylation, we found that removing one copy of the *flacc* allele results in female lethality (Fig. 4E). Likewise, the female-lethal single amino acid substitution allele *vir2F* interferes with Sxl recruitment, resulting in impaired Sxl autoregulation and inclusion of the male-specific exon (Hilfiker et al. 1995). We observed that female lethality of these alleles was rescued by *flacc* and *nito* double heterozygosity, further confirming the involvement of Flacc in *Sxl* alternative splicing (Fig. 4F).

### Zc3h13/Flacc stabilizes Wtap/Fl(2)d–Rbm15/Nito interaction

**Figure 5.**
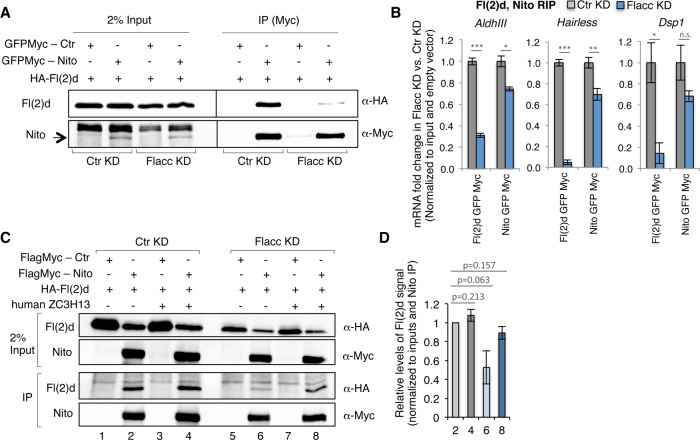
Flacc bridges the methyltransferase complex to mRNA targets via binding to Nito. (*A*) Coimmunoprecipitation experiments were carried out with lysates prepared from S2R^+^ cells transfected with GFPMyc-Nito and Fl(2)d-HA. In control lanes, S2R^+^ cells were transfected with Myc alone and an identical HA-containing protein. Extracts were immunoprecipitated with Myc antibody and immunoblotted using Myc and HA antibodies. Two percent of input was loaded. The same experiment was repeated in Flacc knockdown conditions. Interaction between Nito and Fl(2)d is strongly reduced upon depletion of Flacc. (*B*) Fold enrichment of m^6^A-regulated transcripts (*AldhIII*, *Hairless*, and *Dsp1*) over input in Myc-Fl(2)d and Myc-Nito RIP upon depletion of Flacc or in control conditions. The bar chart shows the mean of three biological replicates. Errors bars indicate SD. (*) *P* < 0.01; (**) *P* < 0.001; (***) *P* < 0.0001; (n.s.) not significant, Student's *t*-test. Loss of Flacc strongly affects Fl(2)d binding and, to a milder extent, binding of Nito to m^6^A-regulated transcripts. (*C*,*D*) Coimmunoprecipitation experiments were carried out with lysates prepared from S2R^+^ cells transfected with either FlagMyc-Nito or Fl(2)d-HA. In control lanes, S2R^+^ cells were transfected with FlagMyc alone and an identical HA-containing protein. Extracts were immunoprecipitated with Flag antibody and immunoblotted using Myc and HA antibodies. Two percent of input was loaded. The same experiment was performed upon depletion of Flacc. Human ZC3H13 was transfected in an identical set of experiments. The interaction between Nito and Fl(2)d is strongly reduced upon loss of Flacc (lane *6*) but can be rescued upon expression of human ZC3H13 protein (lane *8*). Quantification of two replicates is shown in *D*.

To obtain insights into the molecular function of Flacc, we investigated interactions between m^6^A writers in the absence of Flacc. We found previously that knockdown of Fl(2)d diminishes the interaction between Mettl3 and Mettl14 (Lence et al. 2016). Interestingly, we found that this interaction is not affected upon Flacc knockdown (Supplemental Fig. S8A,B). However, we observed that depleting Flacc almost completely abolished the association between Nito and Fl(2)d (Fig. 5A), whereas interactions between Vir and different isoforms of Fl(2)d as well as Vir and Nito were not affected (Supplemental Fig. S8C–F). Interactions between Nito and Mettl3/Mettl14 were also not compromised upon depletion of Flacc (Supplemental Fig. S8G,H). This indicates that Flacc stabilizes the complex and might serve as an adapter that connects the RNA-binding protein Nito to Fl(2)d. If this prediction was true, depletion of Flacc should prevent binding of Fl(2)d, but not Nito, to its mRNA targets. To test this hypothesis, we performed RNA immunoprecipitation (RIP) experiments to monitor the binding of these components to well-characterized m^6^A targets in the presence or absence of Flacc. As shown in Figure 5B, binding of Fl(2)d to *AldhIII*, *Hairless*, and *Dsp1* mRNA was strongly decreased upon Flacc knockdown, whereas Nito binding was only slightly affected. Thus, we conclude that Flacc serves as an adapter between Fl(2)d and the mRNA-recruiting factor RBM15/Nito.

**Figure 6.**
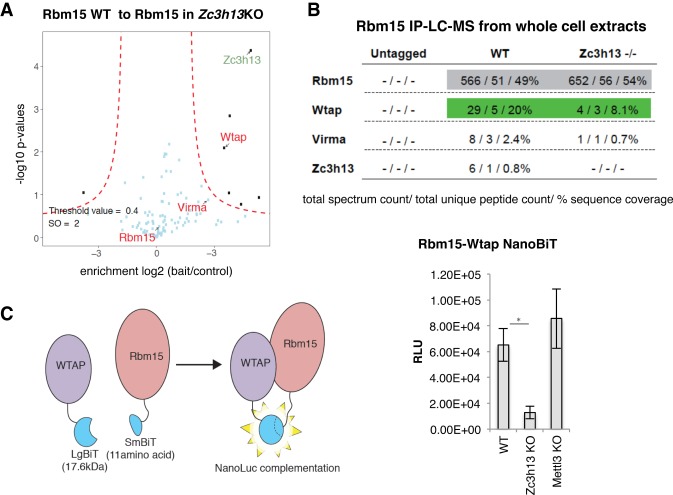
Zc3h13 stabilizes the interaction between RBM15 and WTAP. (*A*,*B*) Comparison of TAP-LC-MS/MS of endogenously Flag-Avi-tagged Rbm15 mESCs in either a wild-type or a *Zc3h13* knockout background. Rbm15 and associated proteins were purified in the presence of 350 mM NaCl. (*A*) Volcano plot showing enriched proteins in wild-type cells (*right*) versus *Zc3h13* knockout cells (*left*). (*B*) Table of spectral counts, unique peptides, and percentage coverage of TAP-LC-MS/MS data in *A*. (*C*) Split luciferase NanoBiT assay examining the interaction of mouse Rbm15 and Wtap. (*Left*) Scheme representing luciferase reconstitution upon transfection of large (LgBit) and small (SmBit) NanoLuc subunit fusions and the interaction of Rbm15 and WTAP. (*Right*) Comparison of Rbm15–Wtap NanoBiT NanoLuc signal in wild-type and *Zc3h13* and *Mettl3* knockout cells. The mean of three independent experiments, three transfections each, is shown. Errors bars indicate SD. (*) *P* = 0.026, calculated using two-tailed Student's *t*-test.

To test functional conservation of Flacc, we cloned a human isoform of ZC3H13 and probed for the interaction between Nito and Fl(2)d upon depletion of endogenous Flacc protein in *Drosophila* S2R^+^ cells. Remarkably, expression of ZC3H13 was sufficient to re-establish the interaction between Nito and Fl(2)d (Fig. 5C,D; Supplemental Fig. S8I–K) even though the two orthologs bear low sequence similarity at the amino acid level (21% identity). These results hint at a conserved role of this newly characterized protein in stabilizing interactions within the MACOM. To address this more directly, we generated *Zc3h13* knockout mESCs that express Flag-Avi-tagged Rbm15 and performed TAP-LC-MS/MS experiments. Starting with both whole-cell extracts and nuclear fractions, Rbm15 interaction with Wtap was markedly reduced (Fig. 6A,B; Supplemental Fig. S9A,B), which is consistent with observations in the fly knockdown experiments [Nito and Fl(2)d, respectively]. Furthermore, the reduced interaction was not attributable to a global decrease of Wtap or other components of MACOM (Supplemental Fig. S9C). As an alternative approach to test MACOM integrity, we used a protein fragment complementation assay (Dixon et al. 2016), generating fusion constructs of Rbm15 and Wtap to NanoBiT subunits. The optimal combination of fusions reconstituted the luciferase signal when transfected into wild-type cells (Wtap N-terminally tagged with the small nanoluciferase subunit and Rbm15 C-terminally tagged with the large nanoluciferase subunit) (Fig. 6C; Supplemental Fig. S9D–F). The relative luciferase signal intensity was strikingly reduced when fusion constructs were transfected in *Zc3h13* knockout but not in *Mettl3* knockout, discarding a secondary effect of global m^6^A loss (Fig. 6C). Taken together, these findings suggest that Zc3h13 acts as an adapter that connects the RNA-binding protein Rbm15 to Wtap also in mammals.

## Discussion

Our study identified a novel interactor of the m^6^A methyltransferase complex, which is conserved in *Drosophila* and mice. Its function in the m^6^A pathway is essential in both species, as its absence results in dramatic reduction of m^6^A levels. The facts that the human homolog was found recently in interactome studies with WTAP (Horiuchi et al. 2013; Wan et al. 2015) and that it can rescue the interaction between Fl(2)d and Nito in *Drosophila* suggest that it has a similar role in human cells. Despite this functional conservation, the protein sequence identity among different homologs is rather weak (Supplemental Fig. S4). Mouse Zc3h13 contains several additional domains as compared with Flacc. In particular, it differs by the presence of a zinc finger domain, which is present in a common ancestor but was lost in dipterian (Supplemental Fig. S4). Other species such as *Ciona intestinalis* also lack the zinc finger motif. In addition, the zinc finger motif can be found in two variants across evolution: one short and one long. As zinc finger motifs are commonly involved in nucleic acid binding or protein–protein interactions, it will be interesting to address the functional importance of this domain when present in the protein. Of note, Zc3h13 appears completely absent in nematodes, as is also the case for Mettl3 (Dezi et al. 2016), possibly indicating that these two proteins have coevolved for the regulation of adenosine methylation.

**Figure 7.**
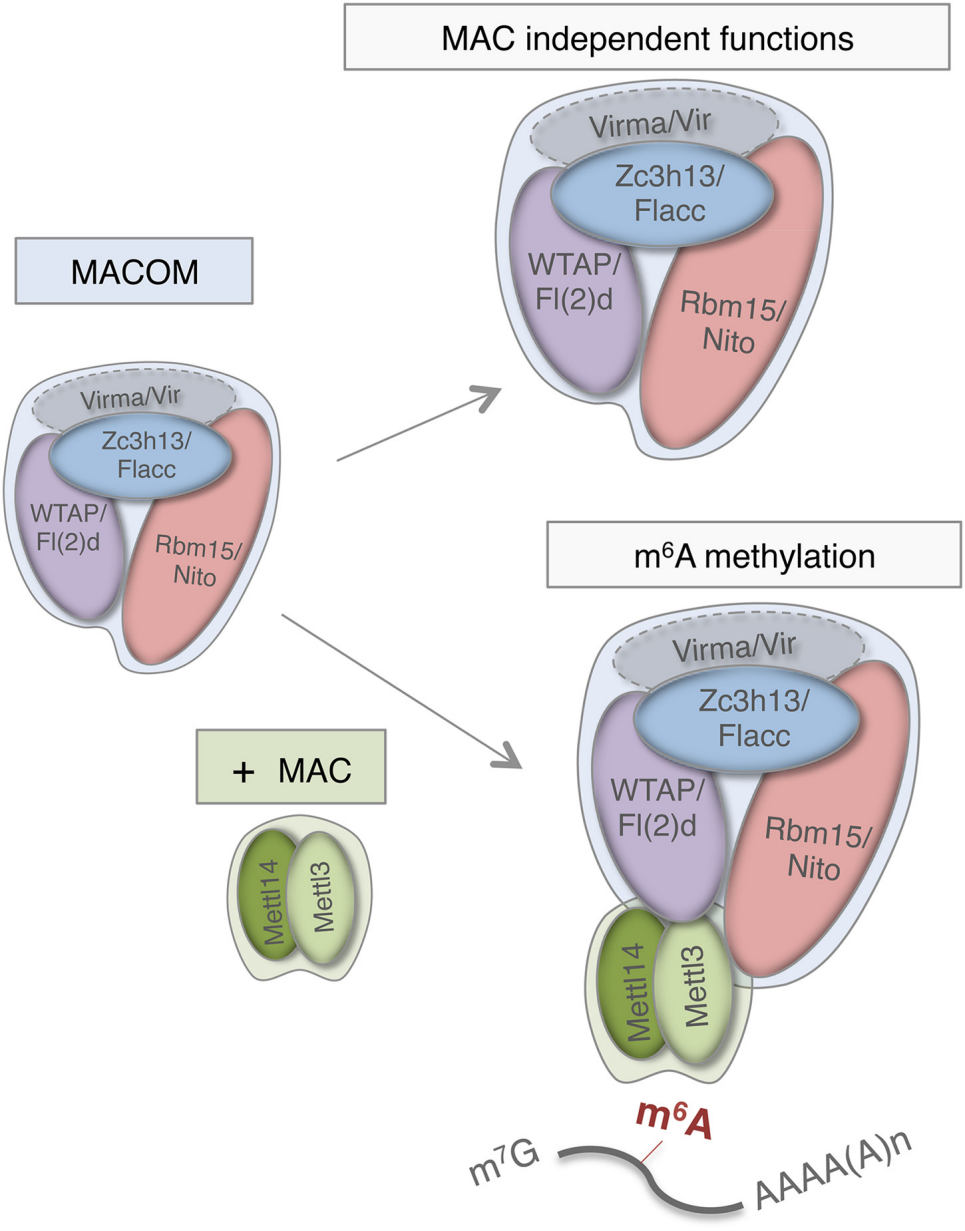
Schematic representation of the role of the MACOM and the MAC. The MACOM can regulate gene expression in two ways: either on its own (MAC-independent functions) or by interacting with MAC components (m^6^A methylation). Flacc (Zc3h13) is a novel component of the MACOM that stabilizes the interaction between Fl(2)d and Nito (Wtap and Rbm15) proteins, thereby ensuring deposition of m^6^A to targeted transcripts.

Our work strongly supports the existence of at least two distinct stable complexes that interact weakly to regulate m^6^A biogenesis. This result is consistent with earlier studies by Rottman and colleagues (Bokar et al. 1997), who isolated two protein components using an in vitro methylation assay and HeLa cell nuclear extracts, which are readily dissociable under nondenaturing conditions. Gel filtration and gradient glycerol sedimentation estimated molecular weights of 200 and 875 kDa (Bokar et al. 1997). While biochemical characterization will be required to address the exact identity of the different complex components, recent biochemical analysis suggests that the 200-kDa complex consists of Mettl3 and Mettl14 (Liu et al. 2014). Although the exact composition of the larger complex is currently unknown, we postulate that it is probably MACOM, consisting of Wtap, Virma, Hakai, Rbm15, and Zc3h13. The calculated total molecular weight of these proteins (600 kDa) is lower than that of the large complex (875 kDa), which suggests the presence of other factors or the inclusion of some subunits in multiple copies. For instance, recombinant WTAP can form aggregates, suggesting the possibility of higher complex organization (Liu et al. 2014). Finally, the existence of two complexes is also supported by our genetic analyses, which show that the knockout of *Mettl3* and *Mettl14* results in viable animals, while loss of function of *fl(2)d*, *vir*, *nito*, and *flacc* is lethal during development. This indicates that the MACOM acts beyond m^6^A methylation via Mettl3 (Fig. 7).

The physiological role of ZC3H13 in human cells has been poorly investigated. Recent reports suggest that mutant ZC3H13 facilitates glioblastoma progression and schizophrenia (Oldmeadow et al. 2014; Chow et al. 2017). It is possible that these diseases originate from misregulation of the m^6^A pathway upon ZC3H13 alteration. For instance, the association of m^6^A with cancer progression, in particular with glioblastoma and acute myeloid leukemia, has been demonstrated recently (Barbieri et al. 2017; Cui et al. 2017; Jaffrey and Kharas 2017; Kwok et al. 2017; Li et al. 2017; Vu et al. 2017; Zhang et al. 2017; Weng et al. 2018). Likewise, m^6^A plays an important role in cortical neurogenesis in the human forebrain (Yoon et al. 2017), a region of the brain that has been associated previously with schizophrenia (Heimer 2000). Hence, future studies should determine whether the role of ZC3H13 in these diseases is connected to its m^6^A-dependent function.

## Materials and methods

### mESC culture and genome editing

mESCs (129xC57Bl/6 genetic background; kindly provided by D. Schübeler of the Friedrich Miescher Institute for Biomedical Research) were cultured on gelatin-coated dishes in mES medium (DMEM; Gibco, 21969-035) supplemented with 15% FBS (Gibco), 1× nonessential amino acids (Gibco), 1 mM sodium pyruvate (Gibco), 2 mM L-glutamine (Gibco), 0.1 mM 2-mercaptoethanol (Sigma), 50 mg/mL penicillin, 80 mg/mL streptomycin, MycoZap prophylactic, and LIF conditioned medium at 37°C in 5% CO_2_. Cultured cells were routinely tested for mycoplasma contamination using the VenorGeM mycoplasma detection kit according to manufacturer's recommendation (Sigma). For endogenous gene tagging using SpCas9-2A-mCherry (Knuckles et al. 2017), Rosa26:BirA-V5-expressing cells (RosaB) were transfected with 2 µg of SpCas9-sgRNA-2A-mCherry and 500 ng of ssODN as a donor when integration was desired. Small guide RNA constructs were generated as described in Knuckles et al. (2017). The ssODNs were synthesized as Ultramers by Integrated DNA Technologies, and their sequences are listed in Supplemental Table 2. All transfections were carried out using Lipofectamine 3000 reagent (Invitrogen). Twenty-four hours after transfection, mCherry-positive edited cells were sorted on a BD FACSAria III cell sorter (Becton Dickinson). mESCs were then sparsely seeded for clonal expansion, and then clones were individually picked, split, and screened by PCR for the desired mutation or integration. For tagging of Mettl3 and Rbm15, clones were subsequently screened by Western blotting using anti Flag (Sigma) or HRP-coupled streptavidin to confirm expression of endogenously tagged proteins. For Zc3h13 knockout lines, two independent single guide RNA (sgRNA) constructs were transfected to target sequences flanking exons 9–10, leading to a frameshift mutation and nonfunctional truncated protein. Deletion was confirmed via Western blotting using an anti-Zc3h13 antibody (Abcam, ab70802). Sequences of small guide RNAs are described in Supplemental Table 2.

### *Drosophila* stocks and genetics

*D. melanogaster w*^*1118*^ was used as the wild-type control. Other fly stocks used were Fl(2)d shRNA (HMC03833; Bloomington *Drosophila* Stock Center [BDSC], 55674), Nito shRNA (HMS00166; obtained from Drosophila RNAi Screening Center [DRSC] [Harvard]), and Flacc dsRNA (GD35212 and KK110253; obtained from Vienna *Drosophila* Resource Center [VDRC]). For genetic interaction studies, we used *Mettl3*^*null*^ (Haussmann et al. 2016), *Mettl14*^*fs*^ (Lence et al. 2016), *Ythdc1*^*MI02006*^ (Bloomington), *fl(2)d*^*2*^ (Bloomington), *vir*^*ts1*^ (kind gift from Jamilla Horabin), *nito*^*1*^ (Yan and Perrimon 2015), and *flacc* mutant allele *CG7358*^*C*^ (Bloomington). To remove *daughterless*, *Df(2L)BSC209* (Bloomington) was used. Driver lines used in this study were *dome*-GAL4 (kind gift from Erika Bach, New York University Langone Medical Center) and *elav*-GAL4 (Bloomington). For the analysis of male-to-female transformations, flies of selected genotypes were chosen randomly.

### *Drosophila* cell line

*Drosophila* S2R^+^ cells were embryonic-derived cells obtained from the *Drosophila* Genomics Resource Center (DGRC; at Indiana University; FlyBase accession FBtc0000150). Mycoplasma contamination was not present (verified by analyzing the RNA sequencing [RNA-seq] data from the cell line).

### Cloning

The plasmids used for immunohistochemistry and coimmunoprecipitation assays in *Drosophila* S2R^+^ cells were constructed by cloning the corresponding cDNA in the pPAC vector (Lence et al. 2016) with an N-terminal Myc tag and the Gateway-based vectors with an N-terminal Flag-Myc tag (pPFMW) as well as a C-terminal HA tag (pPWH) (obtained from the DGRC at Indiana University).

### TAP and MS

One confluent 15-cm dish of mESCs per sample was resuspended in 1 mL of ice-cold TAP lysis buffer (150–500 mM NaCl [depending on the experiment], 20 mM Tris-HCl at pH 7.5, 0.5% NP-40, 1 mM EDTA, 10% glycerol, 1 mM DTT supplemented with protease inhibitor cocktail [Roche]) after 0.25% trypsin/EDTA dissociation and PBS wash. Samples were shaken at 1000 rpm for 30 min at 4°C. Lysate was cleared by centrifugation at maximum speed at 4°C on a tabletop centrifuge. The protein concentration of each sample was determined using Bradford assay (Bio-Rad dye). Equal amounts of lysate (5 mg) from the control sample (parental untagged cells) and the bait protein sample (gene-tagged cells) were normalized by adding an appropriate amount of cold TAP lysis buffer to each sample to adjust the final sample concentration to ∼5 mg/mL. Equilibrated Flag M2 Dynabeads (10 µL of packed bead slurry per 5 mg of protein per sample; Sigma) previously washed twice with 1 mL of cold TAP lysis buffer were added to 5 mg/mL lysate. Tubes containing beads and lysates were incubated overnight at 4°C with end-over-end rotation.

The next day, Dynabeads were washed four times with TAP buffer for 10 min followed by three 15-min elutions of bound proteins with 3xFlag peptide (final concentration of 0.3 mg/mL in TAP buffer; Sigma). Next, elutions were pooled and added to the TAP-washed strepavidin Dynabeads (Thermo Fisher) and incubated overnight at 4°C with rotation. The next day, streptavidin Dynabeads were washed four times with TAP buffer for 10 min followed by a wash with TAP buffer without NP-40. The enriched proteins were digested directly on the Dynabeads with 0.1 mg/mL trypsin in digestion buffer (50 mM Tris at pH 8.0, 1 mM CaCl_2_, 1 mM TCEP).

The generated peptides were acidified with TFA to a final concentration of 0.8% and analyzed by capillary LC-MS/MS with an EASY-nLC 1000 using the two-column setup (Thermo Scientific). The peptides were loaded with 0.1% formic acid and 2% acetonitrile in H_2_O onto a peptide trap (Acclaim PepMap 100, 75 µm × 2 cm, C18, 3 µm, 100 Å) at a constant pressure of 800 bars. Peptides were separated at a flow rate of 150 nL/min with a linear gradient of 2%–6% buffer B (0.1% formic acid in acetonitrile) in buffer A (0.1% formic acid) for 3 min followed by a linear increase from 6% to 22% in 40 min, 22% to 28% in 9 min, 28% to 36% in 8 min, and 36% to 80% in 1 min, and the column was finally washed for 14 min at 80% B on a 50-µm × 15-cm ES801 C18, 2-µm, 100 Å column (Thermo Scientific) mounted on a DPV ion source (New Objective) connected to an Orbitrap Fusion mass spectrometer (Thermo Scientific). The data were acquired using 120,000 resolution for the peptide measurements in the Orbitrap and a top T (3-sec) method with HCD fragmentation for each precursor and fragment measurement in the ion trap according to the recommendations of the manufacturer (Thermo Scientific).

Protein identification and relative quantification of the proteins were done with MaxQuant version 1.5.3.8 using Andromeda as the search engine (Cox et al. 2011) and label-free quantification (LFQ) (Cox et al. 2014) as described in Hubner et al. (2010). The mouse subset of UniProt version 2015_01 combined with the contaminant database from MaxQuant was searched, and the protein and peptide false discovery rates (FDRs) were set to 0.01. All MaxQuant parameters are in the uploaded parameter file mqpar.xml.

Statistical analysis was done in Perseus (version 1.5.2.6) (Cox et al. 2011, 2014; Tyanova et al. 2016). Results were filtered to remove reverse hits, contaminants, and peptides found in only one sample. Missing values were imputed, and potential interactors were determined using *t*-test and visualized by a volcano plot. Significance lines corresponding to a given FDR were determined by a permutation-based method (Tusher et al. 2001). Threshold values (FDR) were selected between 0.005 and 0.05 and SO (curve bend) between 0.2 and 2 and are shown in the corresponding figures. Results were exported from Perseus and visualized using statistical computing language R.

All MS raw data were deposited in ProteomeXchange (data set PXD00811).

### iBAQ analysis

iBAQ was performed as described in Schwanhausser et al. (2011) to evaluate protein abundances in the MAC and the MACOM in Mettl3 TAP-LC-MS experiments.

### m^6^A-RIP

Total RNA from mESCs was isolated using Absolutely RNA Microprep kit (Stratagene) followed by mRNA selection using double Oligo d(T)23 (New England Biolabs) purification. Five micrograms of mRNA was incubated with 4 µg of anti-m^6^A antibody (polyclonal rabbit; Synaptic Systems, catalog no. 202 003) in m^6^A immunoprecipitation buffer (150 mM NaCl, 10 mM Tris-HCl at pH 7.4, 0.1% NP-40) supplemented with 5 U/mL murine RNase inhibitor (New England Biolabs) for 2 h at 4°C. Ten microliters of protein G magnetic beads (Invitrogen) was added to all m^6^A immunoprecipitation samples for 2 h at 4°C. On-bead digestion with RNase T1 (Thermo Fisher) at a final concentration 0.1 U/mL was performed for 15 min at room temperature. Beads with captured RNA fragments were then immediately washed twice with 500 µL of ice-cold m^6^A immunoprecipitation buffer and twice with room temperature m^6^A immunoprecipitation buffer and further eluted with 100 µL of elution buffer (20 mM DTT, 150 mM NaCl, 50 mM Tris-HCl at pH 7.4, 1 mM EDTA, 0.1% SDS, 5 U/mL proteinase K) for 5 min at 42°C. The elution step was repeated four times, and 600 µL of acidic phenol/chloroform (pH 4.5) (Ambion) was added to 400 µL of the combined eluate per sample in order to extract captured RNA fragments. Samples were mixed and transferred to Phase Lock Gel Heavy tubes (5Prime) and centrifuged at 12,000*g* for 5 min. Aqueous phase was precipitated overnight at −80°C. On the following day, samples were centrifuged, washed twice with 80% EtOH, and resuspended in 15 µL of RNase-free H_2_O (Ambion). Recovered RNA was analyzed on RNA Pico Chip (Agilent), and concentrations were determined with RNA HS Qubit reagents. Since no RNA was recovered in the m^6^A immunoprecipitation no-antibody control samples, libraries were prepared with 30 ng of two independent m^6^A immunoprecipitations performed on RNA from wild-type and *Mettl3* and *Zc3h13* knockout cells. For every condition, input material (200 ng of mRNA) was also sequenced. Both m^6^A immunoprecipitations and inputs were sequenced using the NEBNext RNA directional library preparation kit.

### m^6^A RIP sequencing analysis

MACS2 was used to call peaks of m^6^A enrichment for wild-type immunoprecipitation versus input samples using the default parameters. Peaks were assigned to overlapping gene bodies within 500 base pairs (bp). The intersection of the resulting gene lists (3285 genes) was taken as the set of m^6^A target genes.

BigWig files for each sample were created using the qExportWig function from the QuasR package in R (Cox et al. 2011). Read counts were binned in 50-bp windows, and counts for each sample were scaled to the mean aligned library size of all samples. The deepTools suite was used for metagene analysis (Ramirez et al. 2016). The bigwigCompare function was used to calculate the log_2_ ratio between each wild-type or *Zc3h13* knockout sample and the *Mettl3* knockout samples. m^6^A target CDS regions were scaled to 5 kb, and m^6^A enrichment versus *Mettl3* knockout was calculated in 50-bp bins across scaled target regions as well as 2 kb upstream and downstream using the computeMatrix command.

### NanoBiT protein complementation assay

Fusion constructs of mouse Rbm15 and Wtap to NanoBiT subunits were generated as follows: Full-length Rbm15- and Wtap-coding sequences were amplified with the oligonucleotides indicated in Supplemental Table 2 from poly-A-selected mRNA using NEBNext High-Fidelity 2X PCR master mix (New England BioLabs). Overhangs with homology to destination vectors (pBiT1.1-C, pBiT2.1-C, pBiT1.1-N, and pBiTN.1-C; Promega) were included in oligonucleotide sequences. Gel-purified PCR products were cloned into EcoRI sites using NEBuilder HiFi DNA assembly kit (New England BioLabs) following the manufacturer's recommendations. The optimal combinations of N-terminal- or C-terminal-tagged fusions to small or large subunits were determined through transfection of 20,000 wild-type mESCs per well with Lipofectamine 3000 reagent (Invitrogen) seeded in 96-well tissue culture plates (Corning, catalog no. 3917). Measurements were performed using the Nano-Glo live-cell assay system (Promega) and measured in a microplate luminometer (Berthold, LB960). The Rbm15/Wtap fusion combination yielding the highest luciferase activity was then transfected into distinct mESC genetic backgrounds, and the expression level of the fusion construct was quantified via RT-qPCR using oligonucleotides described in Supplemental Table 2.

### *Drosophila* staging

The staging experiment was performed as described previously (Lence et al. 2016) using *D. melanogaster w*^*1118*^ flies. A total of three independent samples was collected for each *Drosophila* stage as well as for heads and ovaries. Samples from the staging experiment were used for RNA extraction to analyze m^6^A abundance in mRNA and expression levels of different transcripts during *Drosophila* development.

### RNA isolation and mRNA purification

Total RNA from S2R^+^ cells was isolated using Trizol reagent (Invitrogen), and DNA was removed with DNase I treatment (New England Biolabs). Fly heads from 3- to 5-d-old flies were separated and homogenized in Trizol prior to RNA isolation. mRNA was isolated by two rounds of purification with Dynabeads Oligo d(T)25 (New England Biolabs).

### RT–PCR

cDNA was prepared using M-MLV reverse transcriptase (Promega). Transcript levels were quantified using Power SYBR Green PCR master mix (Invitrogen) and the oligonucleotides indicated in Supplemental Table 2. RT–PCR was performed using the oligonucleotides described in Supplemental Table 2 to analyze *Sxl* splicing.

### RNA in situ hybridization

In situ hybridization was performed as described previously (Lence et al. 2016). In situ probes were prepared with DIG RNA-labeling kit (Roche) following the manufacturer's protocol. Oligos used for the probes are listed in Supplemental Table 2.

### RIP

S2R^+^ cells were transfected with Myc-tagged constructs using Effectene reagent. Seventy-two hours after transfection, cells were washed with ice-cold PBS and collected by centrifugation at 1000*g* for 5 min. The cell pellet was lysed in 1 mL of lysis buffer (50 mM Tris-HCl at pH 7.4, 150 mM NaCl, 0.05% NP-40) supplemented with protease inhibitors, rotated head over tail for 30 min at 4°C, and centrifuged at 18,000*g* for 10 min at 4°C to remove the remaining cell debris. Protein concentrations were determined using Bradford reagent (Bio-Rad). For RIP, 2 mg of protein was incubated with 2 µg of anti-Myc antibody coupled to protein G magnetic beads (Invitrogen) in lysis buffer and rotated head over tail for 4 h at 4°C. The beads were washed three times with lysis buffer for 5 min. One-fourth of the immunoprecipitated protein–RNA complexes were eluted by incubation in 1× NuPAGE LDS buffer (Thermo Fisher) for 10 min at 70°C for protein analysis. RNA from the remaining protein–RNA complexes was further isolated using Trizol reagent. qPCR was performed with the oligos listed in Supplemental Table 2.

### Immunostaining

For staining of *Drosophila* S2R^+^ cells, cells were transferred to the polylysine-pretreated eight-well chambers (Ibidi) at a density of 2 × 10^5^ cells per well. After 30 min, cells were washed with 1× DPBS (Gibco), fixed with 4% formaldehyde for 10 min, and permeabilized with PBST (0.2% Triton X-100 in PBS) for 15 min. Cells were incubated with mouse anti-Myc (1:2000; Enzo, 9E10) in PBST supplemented with 10% donkey serum overnight at 4°C. Cells were washed three times for 15 min in PBST and then incubated with secondary antibody and 1× DAPI solution in PBST supplemented with 10% donkey serum for 2 h at 4°C. After three 15-min washes in PBST, cells were imaged with a Leica SP5 confocal microscope using a 63× oil immersion objective.

### Western blotting

Proteins were extracted for 30 min on ice, the lysates were centrifuged at 16,000*g* for 5 min at 4°C, and protein concentration in the supernatant was determined using the Bio-Rad protein assay. Protein samples were separated on NuPAGE-Novex Bis-Tris 4%–12% gradient gels (Invitrogen) in MOPS buffer for 40 min at 200 V. Semidry transfer to nitrocellulose membrane (Whatman) was performed for 40 min at 15 V. Membranes were blocked for 30 min in 2% nonfat dry milk and TBS–0.05% Tween 20 (TBST) and incubated with primary antibodies overnight at 4°C (Mettl3 [Protein Tech, 15073], Rbm15 [Abcam, ab70549], Zc3h13 [Abcam, ab70802], Hakai [Aviva Systems Biology, Cbll1 ARP39622], Wtap [Protein Tech, 60188], and Tubulin [Abcam, clone YL1/2]). Signal was detected with corresponding HRP-conjugated secondary antibodies and Immobilon Western Chemiluminiscent HRP substrate (Millipore).

### Cell culture, RNAi, and transfection

*Drosophila* S2R^+^ cells were grown in Schneider's medium (Gibco) supplemented with 10% FBS (Sigma) and 1% penicillin–streptomycin (Sigma). For RNAi experiments, PCR templates were prepared using the oligonucleotides indicated in Supplemental Table 2. dsRNA were prepared using T7 megascript kit (New England Biolabs). dsRNA against the bacterial β-galactosidase gene (lacZ) was used as a control for all RNAi experiments. S2R^+^ cells were seeded at a density of 10^6^ cells per milliliter in serum-free medium, and 7.5 µg of dsRNA was added to 10^6^ cells. After 6 h of cell starvation, serum-supplemented medium was added to the cells. dsRNA treatment was repeated after 48 and 96 h, and cells were collected 24 h after the last treatment. Effectene (Qiagen) was used to transfect vector constructs in all overexpression experiments following the manufacturer's protocol.

### Coimmunoprecipitation assay and Western blot analysis

For the coimmunoprecipitation assay, different combinations of vectors with the indicated tags were cotransfected in S2R^+^ cells. Forty-eight hours after transfection, cells were collected, washed with DPBS, and pelleted by centrifugation at 400*g* for 10 min. The cell pellet was lysed in 1 mL of lysis buffer (50 mM Tris-HCl at pH 7.4, 150 mM NaCl, 0.05% NP-40) supplemented with protease inhibitors and rotated head over tail for 30 min at 4°C. Nuclei were collected by centrifugation at 1000*g* for 10 min at 4°C, resuspended in 300 µL of lysis buffer, and sonicated with five cycles of 30 sec on and 30 sec off at the low power setting. Cytoplasmic and nuclear fractions were joined and centrifuged at 18,000*g* for 10 min at 4°C to remove the remaining cell debris. Protein concentrations were determined using Bradford reagent (Bio-Rad). For immunoprecipitation, 2 mg of proteins was incubated with 2 µg of anti-Myc antibody coupled to protein G magnetic beads (Invitrogen) in lysis buffer and rotated head over tail overnight at 4°C. The beads were washed three times for 15 min with lysis buffer, and immunoprecipitated proteins were eluted by incubation in 1× NuPAGE LDS buffer (Thermo Fisher) for 10 min at 70°C. Eluted immunoprecipitated proteins were removed from the beads, and DTT was added to 10% final volume. Immunoprecipitated proteins and input samples were analyzed by Western blot after incubation for an additional 10 min at 70°C.

For Western blot analysis, proteins were separated on a 7% SDS-PAGE gel and transferred to a nitrocellulose membrane (Bio-Rad). After blocking with 5% milk in 0.05% Tween in PBS for 1 h at room temperature, the membrane was incubated with primary antibody in blocking solution overnight at 4°C. Primary antibodies used were mouse anti-Flag (1:2000; Sigma, M2-F1804), mouse anti-Myc (1:2000; Enzo, 9E10), mouse anti-HA (1:1000; Covance, 16B12), mouse anti-Tubulin (1:2000; Biolegend, 903401), mouse anti-Fl(2)d (1:500; Developmental Studies Hybridoma Bank, 9G2), and rabbit anti-Mettl14 and guinea pig anti-Mettl3 (1:500) (Lence et al. 2016). The membrane was washed three times in PBST for 15 min and incubated for 1 h at room temperature with secondary antibody in blocking solution. Protein bands were detected using SuperSignal West Pico chemiluminescent substrate (Thermo Scientific).

### SILAC experiment and LC-MS/MS analysis

For SILAC experiments, S2R^+^ cells were grown in Schneider medium (Dundee Cell) supplemented with either heavy (Arg8 and Lys8) (Cambridge Isotope Laboratories) or light (Arg0 and Lys0) (Sigma) amino acids. For the forward experiment, Myc-Nito was transfected in heavy-labeled cells, and Myc alone was transfected in light-labeled cells. The reverse experiment was performed vice versa. The coimmunoprecipitation experiment was done as described earlier. Before elution, beads of the heavy and light lysates were combined in a 1:1 ratio and eluted with 1× NuPAGE LDS buffer that was subjected to MS analysis as described previously (Bluhm et al. 2016). Raw files were processed with MaxQuant (version 1.5.2.8) and searched against the UniProt database of annotated *Drosophila* proteins (*D. melanogaster*: 41,850 entries, downloaded January 8, 2015).

### LC-MS/MS analysis of m^6^A levels

Three-hundred nanograms of purified mRNA was digested using 0.3 U of nuclease P1 from *Penicillum citrinum* (Sigma-Aldrich) and 0.1 U of snake venom phosphodiesterase from *Crotalus adamanteus* (Worthington). RNA and enzymes were incubated in 5 mM ammonium acetate (pH 5.3) for 2 h at 37°C. The remaining phosphates were removed by 1 U of FastAP (Thermo Scientific) in a 1-h incubation at 37°C in 10 mM ammonium acetate (pH 8). The resulting nucleoside mix was then spiked with ^13^C stable isotope-labeled nucleoside mix from *Escherichia coli* RNA as an internal standard (SIL-IS) to a final concentration of 6 ng/µL for the sample RNA and 2 ng/µL for the SIL-IS. For analysis, 10 µL of the previously mentioned mixture was injected into the LC-MS/MS machine. Generation of technical triplicates was obligatory. Mouse mRNA samples were analyzed in biological duplicates, and fly samples were analyzed in triplicates. LC separation was performed on an Agilent 1200 series instrument using 5 mM ammonium acetate buffer as solvent A and acetonitrile as buffer B. Each run started with 100% buffer A, which was decreased to 92% within 10 min. Solvent A was further reduced to 60% within another 10 min. Until minute 23 of the run, solvent A was increased to 100% again and kept at 100% for 7 min to re-equilibrate the column (Synergi Fusion; 4 µM particle size, 80 Å pore size, 250 × 2.0 mm; Phenomenex). The ultraviolet signal at 254 nm was recorded via a DAD detector to monitor the main nucleosides.

MS/MS was then conducted on the coupled Agilent 6460 triple-quadrupole (QQQ) mass spectrometer equipped with an Agilent JetStream ESI source that was set to the following parameters: gas temperature, 350°C; gas flow, 8 L/min; nebulizer pressure, 50 psi; sheath gas temperature, 350°C; sheath gas flow, 12 L/min; and capillary voltage, 3000 V. To analyze the mass transitions of the unlabeled m^6^A and all ^13^C m^6^A simultaneously, we used the dynamic multiple reaction monitoring mode. The quantification was conducted as described previously (Kellner et al. 2014).

### RNA-seq and computational analysis

Illumina TruSeq sequencing kit (Illumina) was used for RNA-seq and computational analysis. The RNA libraries were sequenced on a NextSeq500 with a read length of 85 bp. The data were mapped against Ensembl release 90 of *D. melanogaster* using STAR (version 2.5.1b). Counts per gene were derived using featureCounts (version 1.5.1). Differential expression analysis was performed using DESeq2 (version 1.16.1) and filtered for an FDR <5%. Differential splicing analysis was performed using rMATS (version 3.2.5) and filtered for an FDR <10%. Sequencing depth-normalized coverage tracks were generated using Bedtools (version 2.25.0), Samtools (version 1.3.1), and Kentutils (version 302). The heat map of the fold change (log_2_) of commonly misregulated genes was clustered according to rows and columns. The color gradient was adjusted to display the 1% lowest/highest values within the most extreme color (lowest values as the darkest blue and highest values as the darkest red). Splice events for different knockdown conditions are represented by pie charts. “Control” depicts the detected splice events, on average, in all of the comparisons of control versus knockdown. The pie charts for the individual knockdowns depict the amount of significantly different splicing events with a FDR value <10%. The gene ontology (GO) term analysis was performed using the package ClusterProfiler. The GO terms were semantic similarity-reduced using the “simplify” function of the package. The genes tested in all of the conditions were used as a background gene set. Default parameters were used for the analysis. We defined the set of genes that was analyzed for differential expression (in any condition) and whose transcripts contained m^6^A (according to Kan et al. 2017). The significance of the overlap of these genes with the genes commonly differentially regulated in the knockdowns (either commonly up-regulated, commonly down-regulated, or misregulated in all conditions) or commonly misspliced in all conditions was tested using a hypergeometric test.

### Phylogenetic analysis

The phylogenetic tree was constructed with ClustalX from multiple sequence alignments generated with MUSCLE of the *Drosophila* sequence with orthologs from human and other representative species.

### Statistics

For m^6^A level measurements, data sets were compared using two-tailed Student's *t*-test for unequal variances. Normality was verified, and homogeneity of variances was analyzed with Levene's test. RIP-qPCR, ZC3H13 rescue quantification, split luciferase NanoBiT assay, and in vivo Flacc knockdown validation data sets were compared using two-tailed Student's *t*-test for equal variances. Statistical significance of fly viability was determined by a χ^2^ test (GraphPad Prism). Statistical tests used for RNA-seq and m^6^A-RIP data analysis, TAP-MS analysis, iBAQ analysis, and SILAC experiments are described in detail in the relevant sections of the Materials and Methods.

### Data availability

The data that support the findings of this study have been deposited in the NCBI Gene Expression Omnibus (GEO) under accession number GSE106614. All other relevant data are available from the corresponding author.

## Supplementary Material

Supplemental Material
