# Supplementary material for: Zc3h13/Flacc is required for adenosine methylation by bridging the mRNA-binding factor Rbm15/Spenito to the m6A machinery component Wtap/Fl(2)d
Source: Genes Dev. 2018 Mar 1;32(5-6):415–29. doi: 10.1101/gad.309146.117 (PMC5900714; doi:10.1101/gad.309146.117)
Supplement: Supplemental Material [file supp_32_5-6_415__index.html]

Zc3h13/Flacc is required for adenosine methylation by bridging the mRNA-binding factor Rbm15/Spenito to the m6A machinery component Wtap/Fl(2)d — Supplemental Material 

# Zc3h13/Flacc is required for adenosine methylation by bridging the mRNA-binding factor Rbm15/Spenito to the m6A machinery component Wtap/Fl(2)d

## Supplemental Material

- Supplemental\_Table1.xlsx
- Supplemental\_Table2.xlsx
- Supplemental\_Table3.xls
- Supplemental\_Table4.xls
- Supplemental\_Table5.xls
- Supplemental\_Table6.xls
- Supplemental\_Table7.xls
- Supplemental\_Table8.xls
- Supplemental\_Figures.pdf
- Supplemental\_Figure\_Legends.docx
