# Supplementary material for: Zc3h13/Flacc is required for adenosine methylation by bridging the mRNA-binding factor Rbm15/Spenito to the m6A machinery component Wtap/Fl(2)d
Source: Genes Dev. 2018 Mar 1;32(5-6):415–29. doi: 10.1101/gad.309146.117 (PMC5900714; doi:10.1101/gad.309146.117)

Knuckles_Lence309146_FigS1

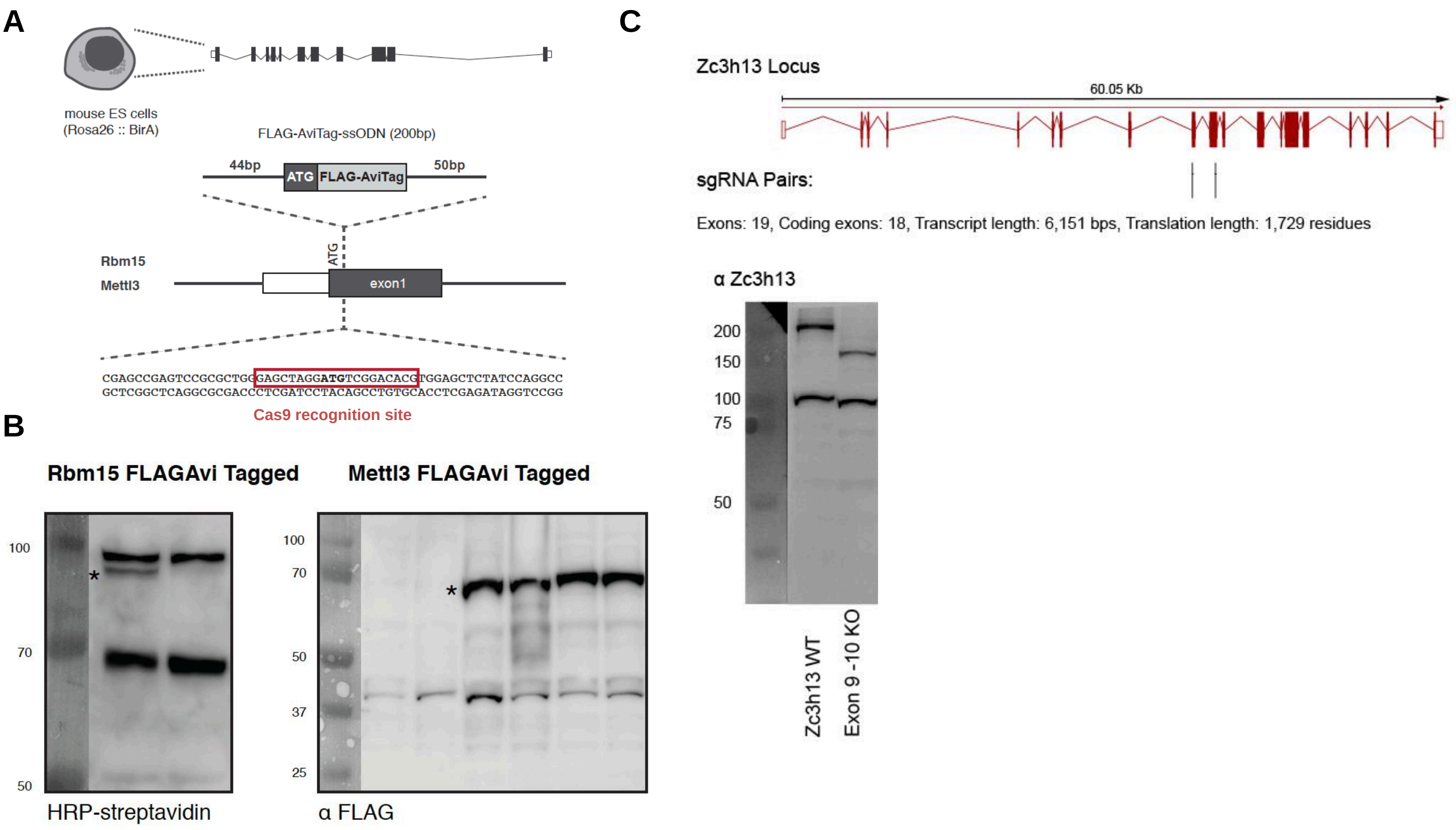

Knuckles_Lence309146_FigS2

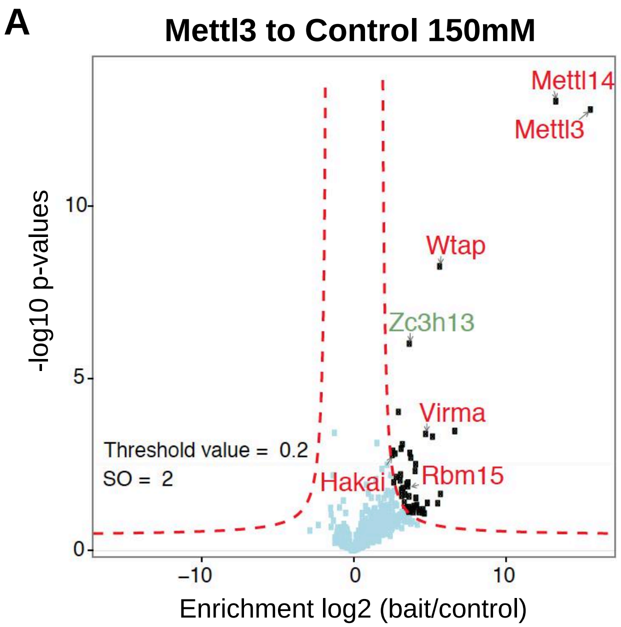

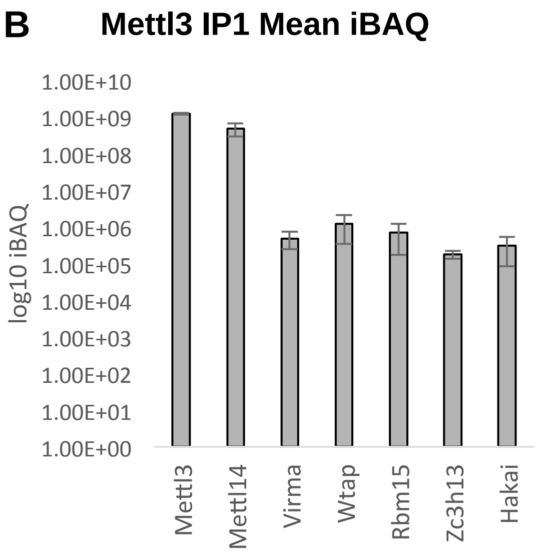

Knuckles_Lence309146_FigS3

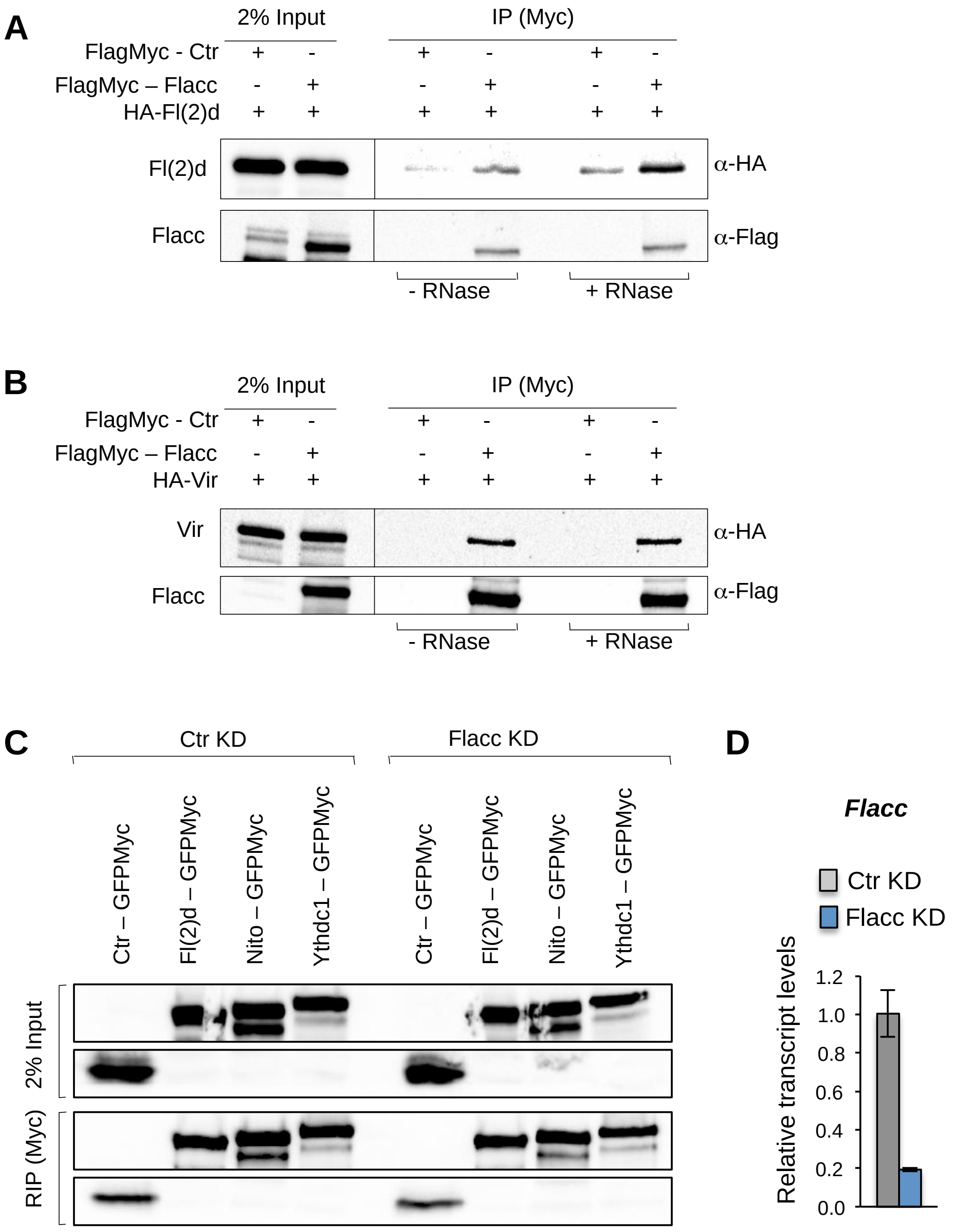

Knuckles_Lence309146_FigS4

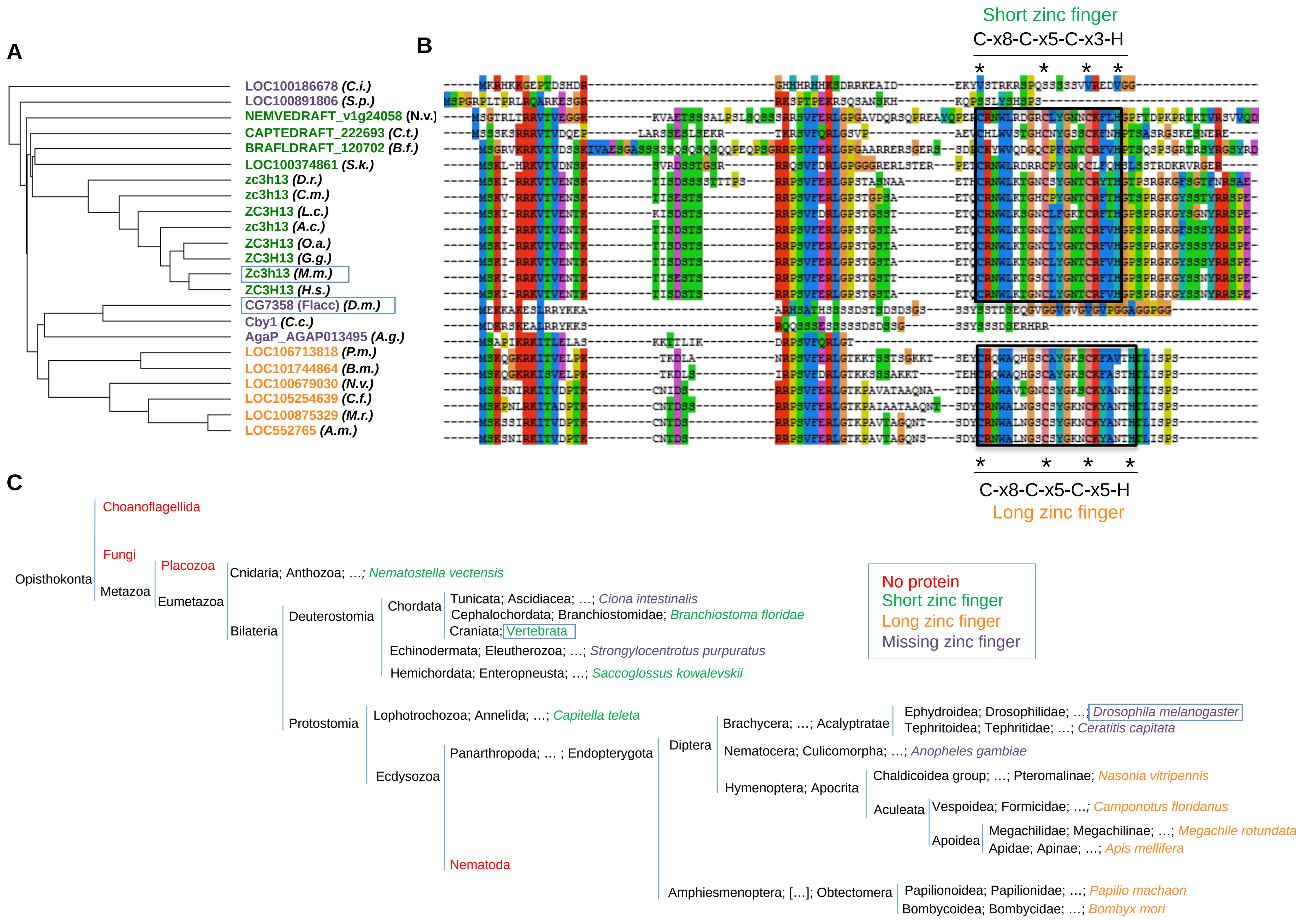

Knuckles_Lence309146_FigS5

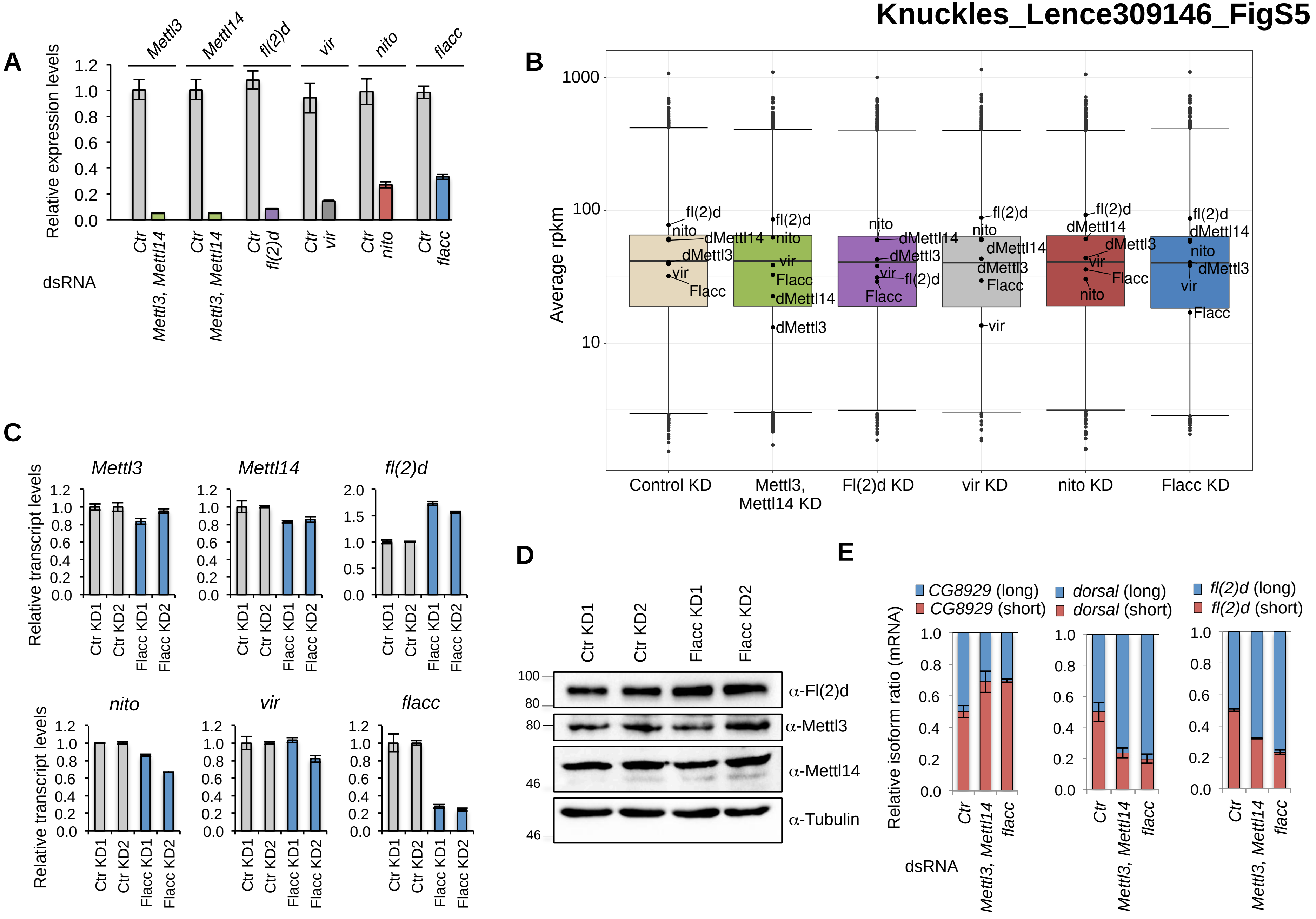

Knuckles_Lence309146_FigS6

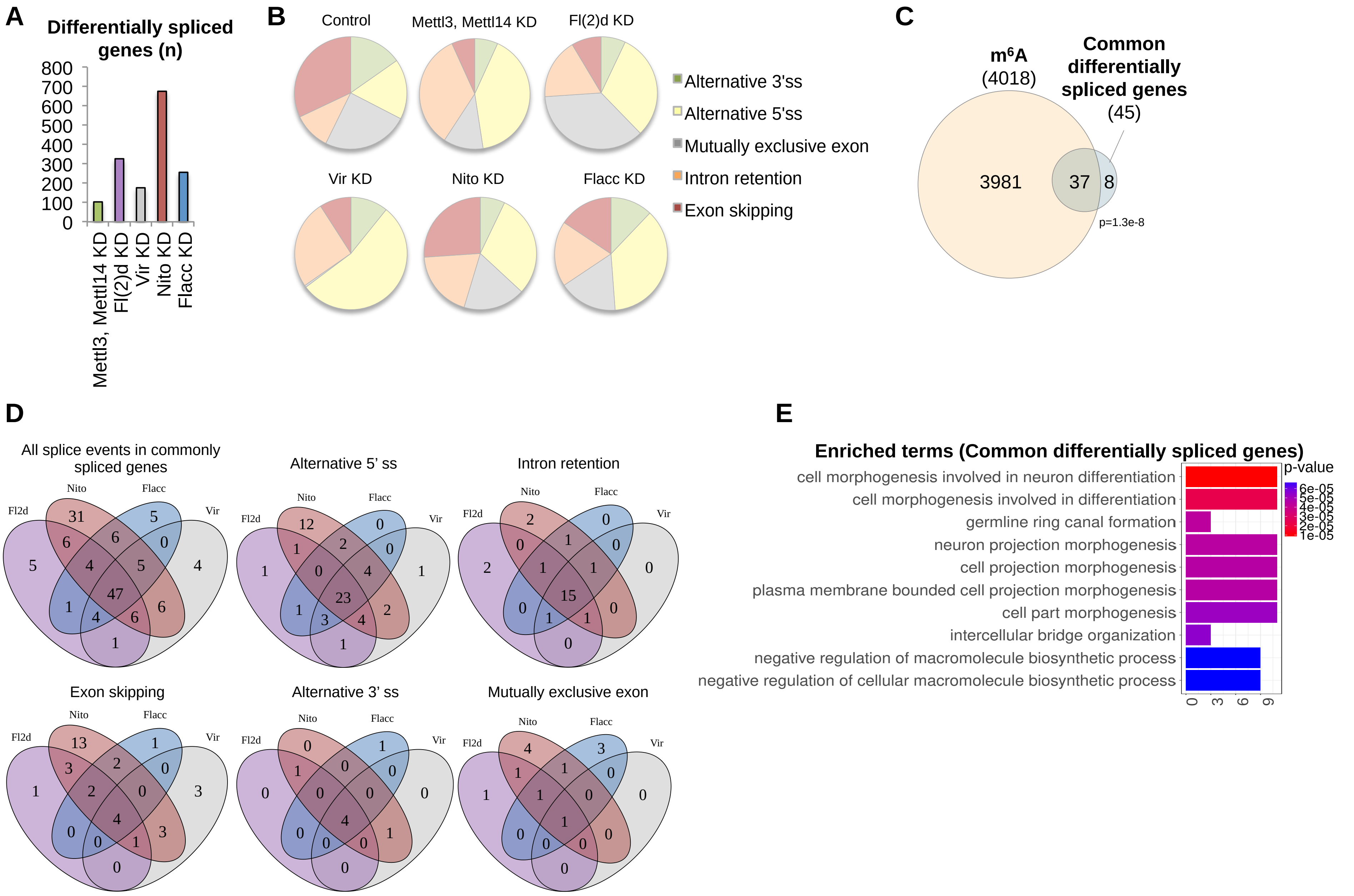

Knuckles_Lence309146_FigS7

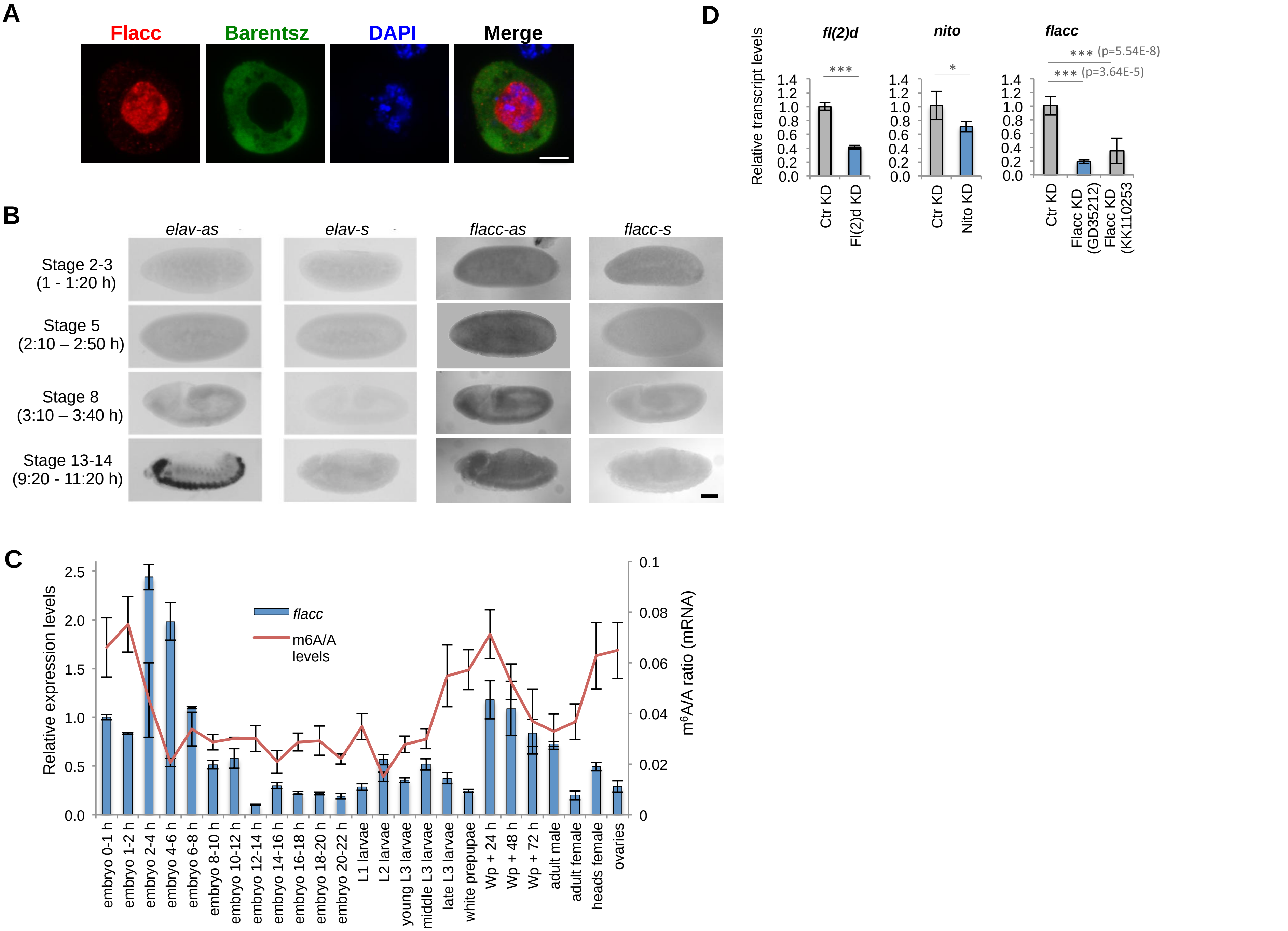

Knuckles_Lence309146_FigS8

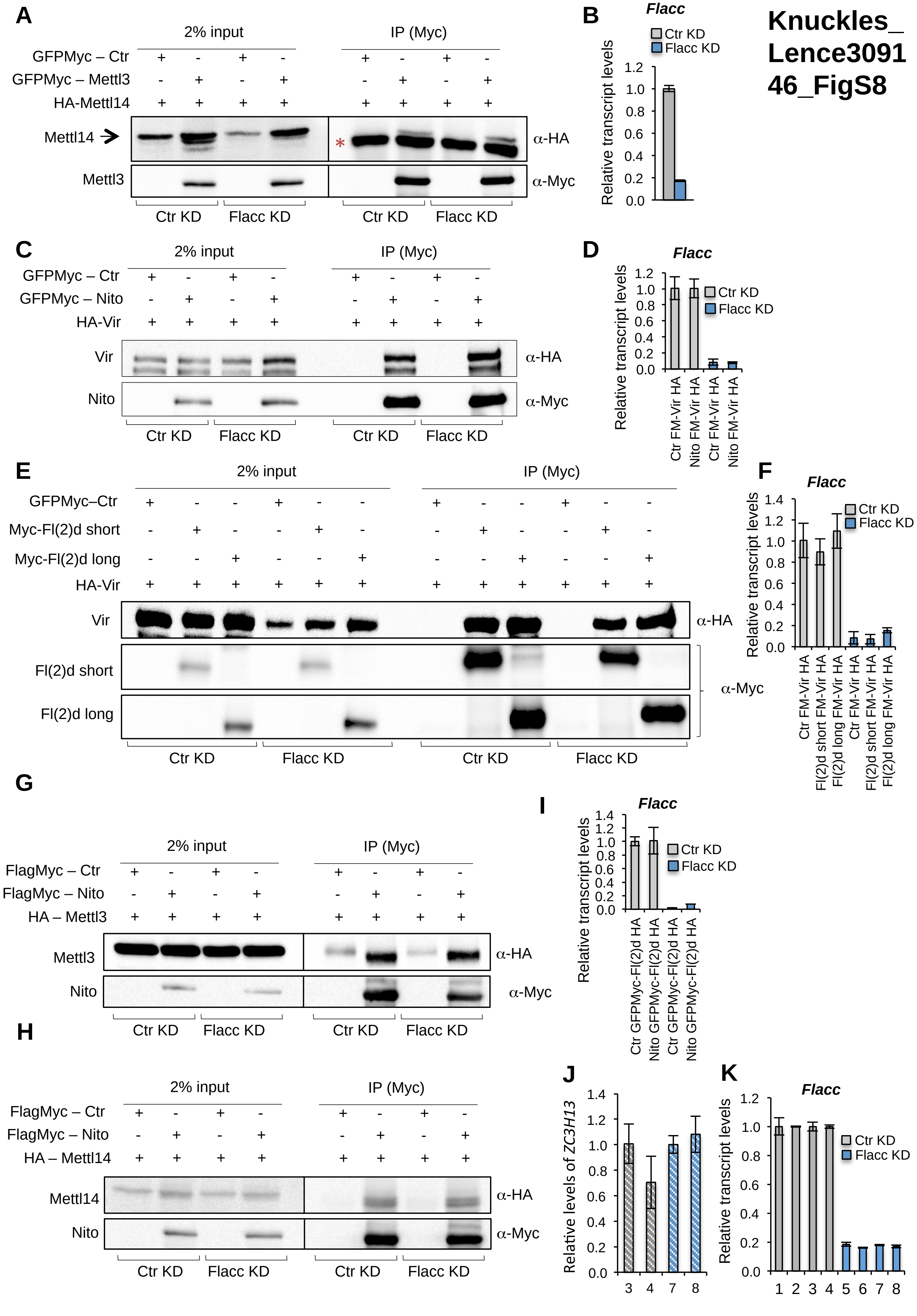

Knuckles_Lence309146_FigS9

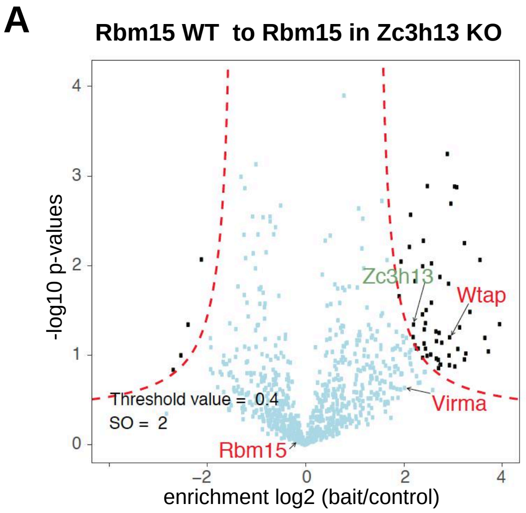

B **Rbm15 IP-LC-MS from nuclear fraction**

	Untagged	WT	Zc3h13 -/-
**Rbm15**	8 / 2 / 3.6%	356 / 27 / 35%	340 / 29 / 30%
**Wtap**	- / - / -	25 / 6 / 20%	1 / 1 / 2.3%
**Virma**	- / - / -	20 / 4 / 3%	1 / 1 / 1.1%
**Zc3h13**	- / - / -	5 / 1 / 1.6%	- / - / -

total spectrum count/ total unique peptide count/ % sequence coverage

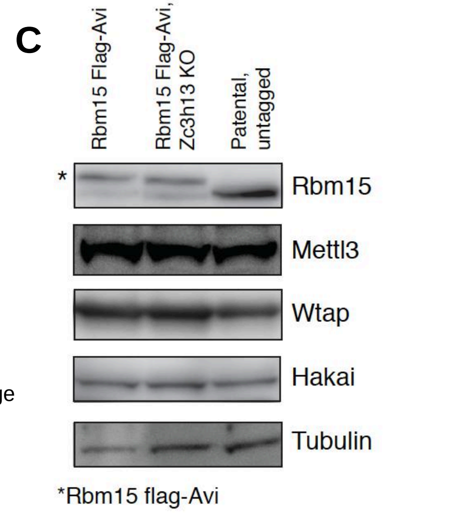

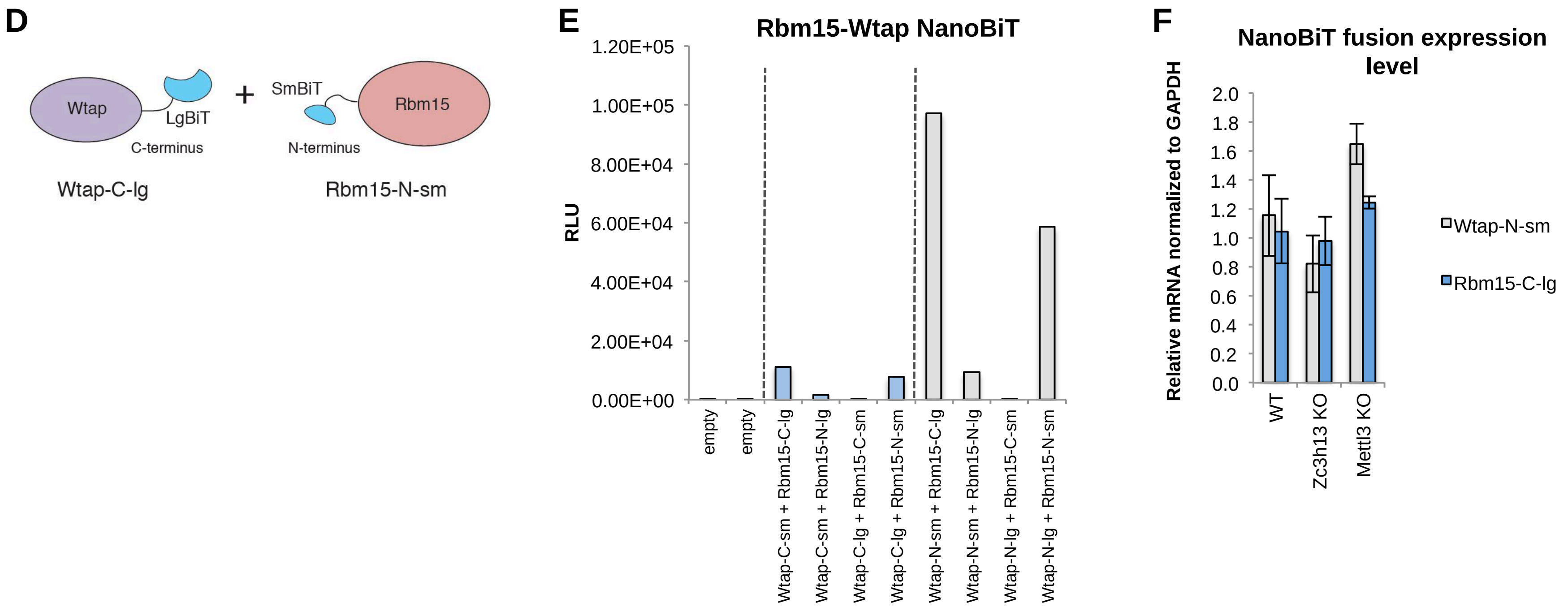

Supplement: Supplemental Material [file supp_gad.309146.117_Supplemental_Figures.pdf]
