# Supplementary material for: Zc3h13/Flacc is required for adenosine methylation by bridging the mRNA-binding factor Rbm15/Spenito to the m6A machinery component Wtap/Fl(2)d
Source: Genes Dev. 2018 Mar 1;32(5-6):415–29. doi: 10.1101/gad.309146.117 (PMC5900714; doi:10.1101/gad.309146.117)
Supplement: Supplemental Material [file supp_gad.309146.117_Supplemental_Figure_Legends.docx]

**Supplemental Figure 1. Generation of tagged and knock-out mESC lines**

**(A)** Schematic of CRISPR/Cas9 strategy to endogenously N-terminally tag Rbm15 and Mettl3 in mESCs expressing the bacterial *BirA* ligase. A ssODN with homology arms was used as donor to integrate the tag. **(B)** Western blot confirmation of clones expressing FLAG-Avi Tagged Rbm15 and Mettl3. For Rbm15, presence of tagged protein was verified by probing a membrane with streptavidin coupled HRP (left); the Mettl3 membrane was probed with anti-Flag antibody as mESCs express an endogenously biotinylated protein of circa. 70 kDa (right). Asterisks mark both tagged proteins, for Mettl3 multiple tagged clones were obtained (lanes 3-6). (**C)** Scheme of CRISPR/Cas9 strategy to ablate Zc3h13. Two independent sgRNAs guide Cas9 to remove exons 9 and 10 resulting in a non-functional truncated protein. Western blot of Zc3h13 to confirm deletion (below).

**Supplemental Figure 2. Flacc/Zc3h13 regulates the m^6^A pathway, supporting data I**

**(A)** TAP-LC-MS/MS of endogenously FLAG-Avi tagged Mettl3 mESC’s compared to Parental untagged in presence of 150mM NaCl. Highlighted are enriched proteins (red) including novel protein Zc3h13 (green). **(B)** iBAQ values for selected proteins for (A).

**Supplemental Figure 3. Flacc/Zc3h13 regulates the m^6^A pathway, supporting data II**

**(A and B)** Co-immunoprecipitation experiments were carried out with lysates prepared from S2R+ cells, transfected with FlagMyc-Flacc and HA-Fl(2)d (A) or HA-Vir (B) . In control lanes, S2R+ cells were transfected with FlagMyc alone and identical HA-containing protein. Extracts were immunoprecipitated with Myc antibody and immunoblotted using Flag and HA antibodies. 2% of input was loaded. The same experiment was repeated in the presence of RNaseT1. Fl(2)d and Vir interact with Flacc in an RNA independent manner. **(C and D)** Western blot validation of RNA immunoprecipitation experiments, which were carried out with lysates prepared from S2R+ cells, transfected with GFPMyc-tagged control, Fl(2)d, Nito and Ythdc1 constructs. Extracts were immunoprecipitated with Myc antibody and immunoblotted using Myc antibody. 2% of input was loaded. The same experiment was performed upon Flacc depletion. Relative expression of *flacc* levels is shown in (D).

**Supplemental Figure 4. Phylogenetic characterization of ZC3H13 proteins.**

(A) Phylogenetic tree of the full sequence alignment of orthologs of ZC3H13 in 23 species. The labels indicate the gene names (from the NCBI’s Entrez database) and the abbreviated species name. (B) N-terminal part of the multiple sequence alignment used to construct the phylogenetic tree, including the zinc-finger (boxes). (C) Tree of the 23 species included in the phylogenetic analyses. Coloring indicates whether the indicated taxa or species contain a ZC3H13 ortholog, and whether the ortholog has the zinc finger or not. The names of 15 species are displayed. The vertebrates included, whose names were not displayed, were: *Danio rerio, Callorhinchus milii, Latimeria chalumnae, Anolis carolinensis, Ornithorhynchus anatinus, Gallus gallus, Mus musculus* and *Homo sapiens.* See methods for details.

**Supplemental Figure 5. Flacc regulates common transcripts with other components of the m^6^A complex, supporting data I**

(A) Relative expression of indicated transcripts upon control (LacZ), Mettl3, Mettl14, Vir, Nito and Flacc KD. The mean standard deviation of three technical measurements from three biological replicates is shown. (B) Boxplots of average expression (rpkm) within replicates for all genes expressed by at least 1 rpkm in the different conditions. The black dots indicate the expresion of m^6^A components in comparison to other expressed genes. (C) Relative expression of indicated transcripts upon control (LacZ) and Flacc KD. (D) WB for Mettl3, Mettl14 and Fl(2)d in control (LacZ) and Flacc KD. (E) (A) Relative isoform quantification of m^6^A-regulated genes (*CG8929, dorsal, fl(2)d*) upon depletion of indicated components. Flacc is required for m^6^A-dependent splicing events.

**Supplemental Figure 6. Flacc regulates common transcripts with other components of the m^6^A complex, supporting data II**

(A) Number of differentially spliced genes upon KD of indicated proteins. (B) Distribution of splicing events in the different KD conditions. The pie chart for “Control” depicts the detected splice events on average in all the comparisons “control vs. KD”. The pie chars for the individual KD depict the amount of significantly different splicing events with a FDR value below 10%. Intron retention and alternative 5`splice site usage are over-represented upon KD of m^6^A components. (C) Overlap between common differentially spliced genes and m^6^A-containing genes (miCLIP data from ([Kan et al. 2017](#_ENREF_1))). The significance of the overlap was tested using a hypergeometric test. Most commonly differentially spliced genes are methylated. (D) Venn diagrams of common differentially spliced events regulated by components of MACOM complex (E) The GO term analysis of common differentially spliced genes, performed using the package ClusterProfiler. Top 10 GO-terms are displayed.

**Supplemental Figure 7**. **Flacc sub-cellular localization and expression through development**

**(A)** Immunostaining of Myc-tagged Flacc protein in S2R+ cells. GFP-tagged Barentsz was used as a cytoplasmic marker. Scale bar, 5 μm. **(B)** *in situ* RNA hybridization of *flacc* (*flacc-as*), *flacc* control (*flacc-s*), *elav* positive control (*elav-as*) and *elav* negative control (*elav-s*) are shown. Scale bars, 100 μm. **(C)** Relative *flacc* mRNA expression and levels of m^6^A in mRNA during *Drosophila* development. Number of hours post-fertilization for different embryo, larval and pupal stages is indicated below. The mean with standard deviation of three technical measurements from three biological replicates is shown. **(D)** Relative expression of indicated transcripts in fly heads upon control, Fl(2)d, Nito or Flacc KD. The mean of three technical measurements from two biological replicates is shown. Errors bars indicate standard deviation (s.d.). *, P<0.01; ***, P<0.0001 (Student’s t-test).

**Supplemental Figure 8. Flacc depletion does not interfere with interactions between two methyltransferases or between Vir and Fl(2)d.**

**(A-F)** Co-immunoprecipitation experiments were carried out with lysates prepared from S2R+ cells, transfected with either GFPMyc-Mettl3 and Mettl14-HA (A), or Myc-Nito and Vir-HA (C), or Myc-Fl(2)d short isoform, Myc-Fl(2)d long isoform and Vir-HA (E). In control lanes, S2R+ cells were transfected with GFPMyc alone and identical HA-containing proteins. Extracts were immunoprecipitated with Myc antibody and immunoblotted using Myc and HA antibodies. 2% of input was loaded. The same experiments were performed upon depletion of Flacc. Interactions between Mettl3 and Mettl14, and Fl(2)d and Vir do not depend on the presence of Flacc (A, C and E). Relative expression of *flacc* levels is shown in (B, D and F). Star indicates IgG band in figure (A).  **(G and H)** Co-immunoprecipitation experiments were carried out with lysates prepared from S2R+ cells, transfected with either FlagMyc-Nito and Mettl3-HA (A), or FlagMyc-Nito and Mettl14-HA (C). In control lanes, S2R+ cells were transfected with GFPMyc alone and identical HA-containing proteins. Extracts were immunoprecipitated with Myc antibody and immunoblotted using Myc and HA antibodies. 2% of input was loaded. The same experiments were performed upon depletion of Flacc. Interactions between Nito and Mettl3/Mettl14 are not affected by depletion of Flacc. **(I)** Related to Figure 5A. Relative levels of *flacc* in indicated samples. The mean with standard deviation of three technical measurements is shown. **(J and K)** Related to Figure 5C. Relative levels of human ZC3H13 transfected in indicated samples (J) and Relative levels of *flacc* in indicated samples (K). The mean with standard deviation of three technical measurements from two biological replicates is shown.

**Supplemental Figure 9. Zc3h13 stabilizes the interaction between Rbm15 and Wtap.**

**(A and B)** Comparison of TAP-LC-MS/MS of endogenously FLAG-Avi tagged Rbm15 mESC’s in either a WT or *Zc3h13* KO background. Rbm15 and associated proteins were purified from nuclear fractions of lysates. (A) Volcano plot showing enriched proteins in WT cells (right) vs *Zc3h13* KO cells. (B) Table of spectral counts, unique peptides and % coverage of TAP-LC-MS/MS data in (A). **(C)** Western blot analysis comparing parental untagged, FLAG-Avi tagged Rbm15 and FLAG-Avi tagged Rbm15 *Zc3h13* KO mESCs levels of Rbm15, Mettl3, Wtap and Hakai, Tubulin was used as loading control). * Denotes shift in Rbm15 due to FLAG-Avi tag. **(D)** Split luciferase NanoBiT assay examining mouse Rbm15 and Wtap. Scheme depicting example fusion constructs generated to determine optimal configuration of fusion proteins. e.g. Wtap-C-lg is C-terminally tagged Wtap fused to the large subunit of NanLuc. **(E)** Luciferase assays comparing all possible LgBit and SmBit NanoLuc subunit fusions to Rbm15 and Wtap to determine which combination provides the strongest signal. **(F)** RT-qPCR measurements of Wtap-N-sm and Rbm15-C-lg fusion constructs in WT, *Zc3h13* KO and *Mettl3* KO backgrounds.

Kan L, Grozhik AV, Vedanayagam J, Patil DP, Pang N, Lim KS, Huang YC, Joseph B, Lin CJ, Despic V et al. 2017. The m(6)A pathway facilitates sex determination in Drosophila. *Nature communications* **8**: 15737.
